# Immune-challenged wild mallards move less and spend more time in sheltered habitats

**DOI:** 10.1186/s40462-026-00715-0

**Published:** 2026-10-22

**Authors:** Elinor Jax, Inge Mueller, Dennis Horvath, Julia Koerner, Wolfgang Fiedler, Daniel Zuñiga, Elena Werner, Roman T. Kellenberger, L. Michael Romero, Marcus Groettrup, Martin Wikelski, Robert H. S. Kraus

**Affiliations:** 1 Department of Migration, Max Planck Institute of Animal Behavior, Radolfzell, Germany; 2 Department of Biology, University of Konstanz, Konstanz, Germany; 3 Crop Genome Dynamics Group, Agroscope, Nyon, Switzerland; 4 Department of Biology, Tufts University, Medford, MA United States of America; 5 Institute of Cell Biology and Immunology Thurgau (BITG) at the University of Konstanz, Kreuzlingen, Switzerland; 6 Department of Biology and Environmental Science, Linnaeus University, Kalmar, Sweden

**Keywords:** Sickness behaviour, Accelerometer tag, GPS tracker, Eco-immunology, Wildlife-disease ecology, Waterfowl

## Abstract

**Background:**

Sickness behaviour is an important component of the response to disease and can influence how animals move through and use their environment. Such changes in behaviour may have consequences for animal ecology and, in species that serve as important hosts for infectious diseases, potentially affect disease dynamics. However, studying sickness behaviour in free-ranging animals remains challenging because it requires identifying when animals are sick while simultaneously tracking their behaviour in their natural environment.

**Methods:**

To improve our understanding of sickness behaviour in wildlife, we investigated sickness-related behavioural responses in wild mallards (*Anas platyrhynchos*). For this purpose, we experimentally induced an acute immune response in free-ranging mallards using lipopolysaccharide (LPS) encapsulated into biodegradable microspheres, and monitored movement patterns and habitat use for one week using GPS and accelerometry bio-loggers.

**Results:**

We show that LPS-treated birds reduced their speed, flew shorter distances, and decreased their overall activity levels compared to control individuals, consistent with lethargy-like sickness behaviour. Furthermore, LPS-treated birds spent more time in sheltered areas and less time on open water during the peak of the acute phase response, with a potential shift in the opposite direction towards the end of the study.

**Discussion:**

These findings improve our understanding of sickness behaviour in free-ranging wildlife and provide a basis for future studies investigating how sickness-related changes in movement and habitat use may affect ecological interactions and disease dynamics under natural host–pathogen contexts.

**Supplementary Information:**

The online version contains supplementary material available at https://doi.org/10.1186/s40462-026-00715-0.

## Background

Sickness behaviour is a coordinated suite of behavioural changes induced by immune activation and includes responses such as lethargy, reduced activity and increased resting [1, 2]. Such changes can alter how animals move through and use their environment, potentially affecting resource acquisition, habitat use and social interactions [3–5]. Because movement also shapes opportunities for contact with conspecifics, other species and pathogens, sickness-induced changes in movement may also have consequences for pathogen transmission [6–8]. Characterising sickness behaviour in free-ranging animals is therefore important for understanding how immune activation can influence animal ecology and potentially affect disease dynamics.

Recent advances in bio-logging technologies have made it possible to track wild individuals while simultaneously recording changes in behaviour [9, 10]. This provides an opportunity to quantify behavioural responses associated with sickness while animals remain in their natural environment. Animal-borne devices measuring GPS (Global Positioning System) and acceleration (ACC) data enable the quantification of behavioural metrics relevant to sickness behaviour, including fine-scale behaviours, such as resting, flying and feeding, as well as broader movement patterns, including migration [11, 12]. Combined with remote sensing data, these approaches also allow detailed assessments of habitat use. Despite these advances, identifying sickness behaviour in free-ranging animals remains challenging because the onset and duration of naturally occurring infections and associated sickness responses are rarely known. Consequently, it remains difficult to link observed behavioural changes in wild animals to underlying sickness responses.

To overcome this challenge, experimental approaches that induce a controlled immune response while allowing animals to remain in their natural environment provide a means of investigating sickness behaviour in free-ranging animals. One such approach is the use of endotoxins such as lipopolysaccharide (LPS), which activates inflammatory pathways and induces an acute phase response without exposing animals to live pathogens [13]. LPS has commonly been used to test hypotheses relating to sickness behaviour in captive animals, including hens and ducks [14, 15], and has also been used to experimentally investigate behavioural responses to immune activation in free-ranging passerines [16–20]. Although LPS does not reproduce all aspects of natural infection, it provides a controlled approach for quantifying behavioural responses associated with acute immune activation.

The mallard (*Anas platyrhynchos*) is one of the world’s most widespread and abundant waterfowl species and exhibits movement patterns ranging from local daily movements to long-distance migration [21–23]. The mallard is also a key natural host of avian influenza viruses (AIV) and has consequently been extensively studied in the context of wildlife disease ecology [24–26]. Its broad distribution, diverse movement strategies, and suitability for carrying high-resolution biologging devices make the mallard a valuable model for investigating sickness behaviour in free-ranging birds. Despite this, important questions remain regarding how sickness behaviour manifests in wild mallards. Observational studies of naturally infected mallards, particularly those infected with low pathogenic avian influenza virus (LPAIV), provide valuable insights into how infection affects behaviour under natural conditions, although reported effects on movement are variable [27–31]. Some studies found no detectable effects of LPAIV infection on movement patterns in free-ranging mallards [27, 28], whereas others reported weak negative associations between LPAIV infection and daily distance travelled in adult males [29] or effects of viral load on stopover duration in migrating juveniles [30]. LPAIV generally causes little or no overt disease in mallards [32–35], and these studies may therefore not capture behavioural responses associated with more pronounced sickness. Such responses may nevertheless be ecologically relevant, as the intensity of sickness responses can vary among infections and host–pathogen combinations. Previous work in captive mallards has characterised LPS-induced immune activation across physiological, cellular and transcriptional levels, including changes in body temperature, heart rate, leukocyte composition, and expression of genes and biological pathways associated with immune and inflammatory responses [36]. Experimental induction of an acute immune response with LPS therefore provides a complementary approach for investigating sickness behaviour in free-ranging mallards, enabling the quantification of sickness-related behavioural responses under a controlled immune challenge.

Here, we investigated short-term behavioural responses associated with sickness behaviour in wild mallards. We used GPS and accelerometry bio-loggers to quantify movement, activity and habitat use in free-ranging mallards following experimental induction of an acute immune response using microsphere-encapsulated lipopolysaccharide (LPS). Specifically, we examined whether immune activation was associated with changes in (1) spatial movement, including the distances travelled and speeds of movement (2), fine-scale activity and movement, as quantified from accelerometry data, and (3) habitat use. Based on sickness behaviour theory, which predicts reduced activity and altered space use during the acute phase response [1, 37–39], we expected LPS-treated birds to show reduced movement and activity compared with controls. We additionally examined whether immune activation was associated with changes in habitat use, including a potential shift towards more sheltered habitats. By experimentally inducing an acute immune response while monitoring free-ranging individuals with high-resolution biologgers, this study provides insight into how sickness behaviour manifests in the movement ecology of wild mallards.

## Methods

### Study system

To investigate sickness behaviour under natural conditions, we captured wild mallards (*Anas platyrhynchos*) in a baited trap at a pond next to the Max Planck Institute of Animal Behavior in Möggingen (Germany: 47°45’59.1”N 8°59’43.6”E) in January and February 2019. Upon capture, all individuals were ringed, weighed and sexed, and oral and cloacal swabs were collected to screen for avian influenza virus, to ensure that only uninfected birds were included. All individuals tested negative (Text S1).

A total of 25 mallards were included in the study. Five birds captured in January 2019 were included in a pilot study aimed at determining the amount of microsphere-encapsulated endotoxins required to induce a prolonged physiological response and assessing how long body temperature remained elevated. The remaining 20 birds were included in the main study, which aimed at investigating behaviour in free-living, immune-challenged mallards. The original study design aimed for a balanced representation of sexes; however, fewer birds than anticipated were captured during the field season, and the majority of captured birds were males, resulting in a male-biased final sample. The pilot study therefore included five males, and each main study group included eight males and two females. However, one logger from each treatment group failed to transmit any data, such that only one female per treatment contributed data to the final analyses.

### Immune challenge

To study sickness behaviour in wild mallards, we experimentally induced an acute phase immune response using the endotoxin LPS. When administered intramuscularly in aqueous solution, LPS induces a rapid, short febrile response (< 24 h) in mallards and Pekin ducks (*Anas platyrhynchos domestica*) [15, 36, 40]. To extend the period of immune stimulation and reduce the influence of capture- and release-related effects on behavioural measurements (as in [16, 17, 41]), we administered LPS encapsulated in depot-forming, biodegradable poly(lactic-co-glycolic acid) (PLGA) microspheres. These microspheres gradually release the embedded agent as the polymer degrades [42, 43], and we expected this formulation to prolong immune stimulation over several days. Such a prolonged response could provide a longer temporal window for studying behavioural changes under field conditions. Still, the formulation was not intended to reproduce the infection dynamics of any specific pathogen.

#### Immune-stimulant preparation

We initially planned to use two non-infectious immuno-stimulants to induce distinct types of innate immune activation associated with bacterial- and viral-like stimuli: LPS (L2630, phenol-extracted Escherichia coli 0111:B4; Sigma-Aldrich) and polyinosinic: polycytidylic acid (poly I:C, P9582, double-stranded homopolymer polyinosinic-polycytidylic acid potassium salt; Sigma-Aldrich). Both were encapsulated in biodegradable PLGA-microspheres composed of a 50:50 mixture of lactide: glycolide (Resomer RG 502 H, Evonik), to achieve a controlled, slow release. The PLGA polymer is FDA (Food and Drug Administration) approved for human applications and is regularly used for slow and controlled drug delivery [44]. In vitro tests showed that 1 mg of microspheres contained approximately 5 µg LPS or 50 µg poly I:C. Released LPS was quantified using a ToxinSensor™ Chromogenic LAL Endotoxin Assay (#L00350, Genscript; Fig. S1).

#### Pilot study

As LPS and poly I:C encapsulated in biodegradable PLGA microspheres had not previously been administered to mallards, we conducted a pilot study to determine effective dosages and to assess whether the microsphere formulation produced a sustained physiological response, as indicated by an extended elevation in body temperature. Dosages were guided by a previous study where LPS and poly I:C were administered to mallards intramuscularly in an aqueous solution [36], and in vitro microsphere release kinetics (Fig. S1).

We injected body temperature–recording passive integrated transponder (PIT) tags (BioThermo13, Biomark Inc., Boise ID, USA) subcutaneously between the wings of five male mallards and administered one of four treatments subcutaneously in the inguinal fold using a 22-gauge needle: 300 µg LPS/kg body weight (low dosage, 60 mg microspheres in 3 mL PBS [phosphate-buffered saline]), 900 µg LPS/kg (high dosage, 180 mg microspheres in 6 mL PBS), 3 mg poly I:C/kg (low dosage, 60 mg microspheres in 3 mL PBS), or 9 mg poly I:C/kg (high dosage, 180 mg microspheres in 6 mL PBS). We injected the fifth bird with empty microspheres (180 mg in 6 mL PBS) as a control.

We housed the mallards individually in aviaries at the Max Planck Institute for Animal Behavior in Radolfzell, Germany, for 72 h post immune challenge. Each aviary compartment (3 × 4 × 2.5 m) included a water basin and a shelter with nesting material, with food provided *ad libitum*. We recorded body temperature every two hours using an external PIT tag reader (HPR Plus, Biomark Inc., Boise ID, USA). After 72 h we examined the health of the birds and released them at the capture site.

The pilot study showed that the high LPS dosage elicited a strong and sustained immune response, with peak body temperature reaching 42.5 °C on the first day and remaining elevated (> 41 °C) during days 2–3 (Fig. S2). In contrast, the average body temperature of the control individual was 40.47 ± 0.24 °C (mean ± SD). Based on these results, 900 µg LPS/kg body weight encapsulated in 180 mg PLGA microspheres was used for the main study. Neither poly I:C dosage elicited a clear temperature response (Fig. S2), and this treatment was therefore excluded from the main study.

#### Main study

**Fig. 1:**
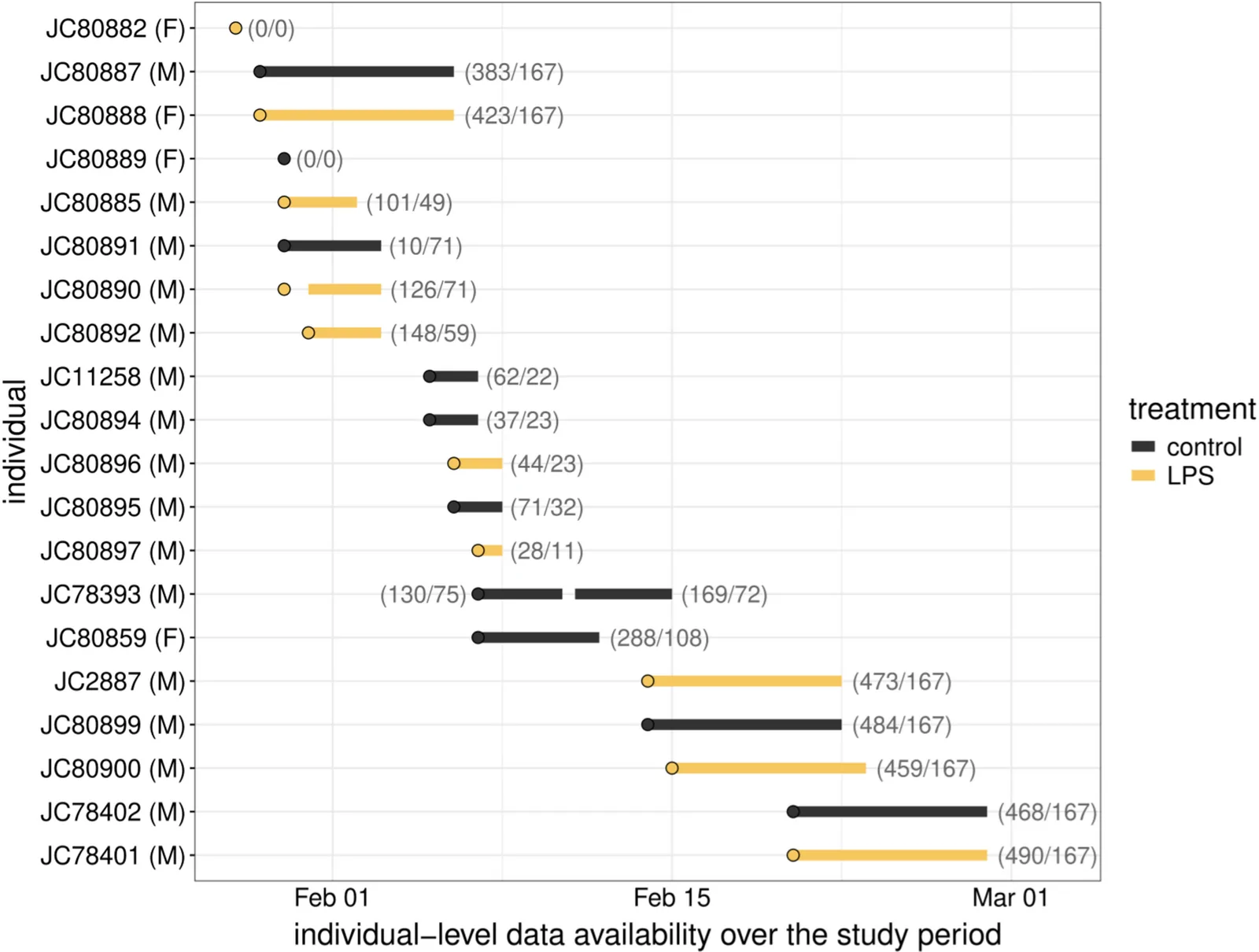
Overview of study inclusion period and logger data availability for each individual included in the main study. Sex and treatment group are indicated for each individual. Dots represent the timing of capture and treatment, while lines indicate the period during which each logger transmitted data (up to seven days post-treatment). Numbers in parentheses show the total number of high-quality GPS fixes (left) and the number of hours with aggregated ACC data (right) available for each individual. The timing of capture and treatment is shown for two loggers that failed to transmit data. Individual JC78393 was recaptured during the study period and equipped with a fully charged replacement logger. Therefore, the number of GPS fixes and aggregated ACC records are presented separately for each logger deployment. For more details, see Table S1

In the main study, we used GPS and three-dimensional accelerometers to track movement and behaviour in free-living mallards after the immune challenge. Twenty birds, captured between 28 January and 20 February 2019 (Fig. 1 and Table S1), were equipped with 25 g GPS-GSM (Global System for Mobile Communications) transmitters with elevated solar panels (OrniTrack-25, Ornitela) using customised backpacks [45]. Based on the pilot results, ten birds (eight males, two females) received the LPS treatment (900 µg LPS/kg body weight, 180 mg MS diluted in 6 mL PBS) and ten (eight males, two females) received empty microspheres as controls (180 mg empty microspheres diluted in 6 mL PBS). We administered the treatments sequentially where possible to reduce the influence of capture date. All individuals were released at the capture site within one hour of capture and 15 min after the treatment.

### Movement patterns and habitat use in free living mallards

#### Data collection

We used bio-loggers to observe changes in behaviour and habitat use in wild mallards during the acute phase response. The loggers were set to record a GPS-fix every 20 min (when the battery level was 50–100%), 40 min (25–49%), or 4 h (< 25%), and 24 acceleration measures in each of the three dimensions per second for five seconds every five minutes. The loggers sent data remotely once per day using the GSM net. We extracted GPS and acceleration data for each individual for 7 days post stimulation (Fig. 1 and Table S1). We excluded data collected within one hour of release to minimise the effect of handling on the data. Furthermore, we only used GPS points with a horizontal dilution of precision (HDOP) of ≤ 2, as they are considered most accurate in their location estimates [46, 47].

We experienced technical issues with the bio-loggers: two devices failed to transmit data entirely, and several of the remaining loggers experienced rapidly decreasing battery levels and therefore sent data less frequently than initially planned or stopped working during the study period (Fig. 1 and S3, Table S1). As a result, data was transmitted from 18 individuals on the first day post stimulation (9 LPS treatment and 9 control individuals) and from eight individuals (4 LPS treatment and 4 control individuals) on the last day of the study period. The analyses thus contain data from different number of individuals depending on how many loggers were still operational at the time. In total, 25,160 acceleration bursts and 4394 GPS fixes with informative positional information and HDOP ≤ 2 were recorded from the 18 bio-loggers during seven days post release. Overall, 92.0% of the GPS fixes were taken at 20-min intervals (18–22 min apart), 7.1% at 40-min intervals (38–42 min apart), 0.05% at 4 h intervals (238–242 min apart) and the remaining fixes outside these ranges (min 4 and max 121 min apart) (Fig. S4).

#### Data analysis

##### Movement patterns

Lethargy is a common sickness behaviour in animals during the acute phase response [1, 48], so we monitored activity levels using high definition accelerometers and calculated the speed and distance between consecutive GPS points (step length) for all individuals during one week post stimulation. We estimated the activity level by calculating the mean of the variance of the acceleration measurements from all axes for each five-second burst, following Korner et al. [49]. We then calculated the mean activity per hour for each individual, leading to a total of 1726 activity measures.

To investigate whether mallards differed in spatial movement patterns, we quantified movement using two metrics: (1) speed, calculated as distance per unit time and (2) distance travelled between consecutive GPS locations (step length). The speed metric was particularly useful for later time point during the study, i.e., when GPS-fixes were transmitted less frequently due to low battery of the loggers. To minimise bias caused by missing data and irregular sampling intervals when calculating the step length, we excluded intervals shorter than 18 min or longer than 22 when estimating the distance metric. We calculated the speed as well as step length using the move package [50] in R version 3.6.1 [51]. To ensure the quality of the tracking data, we excluded GPS fixes with a HDOP value greater than 2 [46, 47]. To account for diurnal variation in movement, each data point was assigned to one of 26 day periods based on local sunrise, sunset, and twilight times (e.g., astronomical, nautical, civil twilight, daytime, night-time) following [49].

We then fitted Generalized Additive Mixed Models (GAMMs) to the movement data in R using the mgcv v. 1.9-3 [52] package. Smooth terms were represented by penalized regression splines, and random effects and autocorrelation were incorporated using gamm from the nlme v. 3.1–168 package [53, 54], with models fitted using maximum likelihood [55]. For each model, hours post-stimulation (hrs_ps) was modelled as a smooth term, with separate smooths estimated for each treatment group using s(hrs_ps, by = treatment) to allow for potentially non-linear and treatment-specific changes over time. Treatment and day period (tz2) were included as fixed effects. We included individual as a random intercept to account for repeated measurements from the same individual, and used the corAR1 function in nlme [53] to account for autocorrelation of sampling points across time. We estimated model-based means and associated 95% confidence intervals (CI) [56] using the predict function in *mgcv*, with uncertainty derived from the covariance matrix of the fitted GAMM parameters. To facilitate interpretation of the model predictions, periods during which the 95% confidence interval of the treatment prediction did not include the estimated mean of the control prediction were interpreted as indicating clear graphical separation between the predicted treatment and control trajectories. This criterion was used solely to aid interpretation of the fitted model and was not treated as a formal statistical hypothesis test.

As a sensitivity analysis, we repeated all GAMMs with sex included as an additional fixed effect to assess whether the observed treatment effects were robust to potential differences in movement behaviour between sexes.

##### Habitat use

Sick individuals may use their habitat differently for several reasons, for example due to predator avoidance or reduced motivation or ability to seek food and water [1, 2, 57]. We were particularly interested in whether the immune response influenced mallards’ use of sheltered habitats near water, such as reed beds and overhanging trees, or the likelihood of visiting sites where humans might provide food. Because publicly available geospatial datasets lacked sufficient habitat resolution, we obtained aerial imagery of the areas used by the mallards and manually digitized land-cover maps, including detailed delineation of sheltered habitats at the time of the study. Using GPS data, we then determined which habitat types were most frequently used by the mallards during the seven days following stimulation and compared habitat use between the treatment groups.

**Fig. 2:**
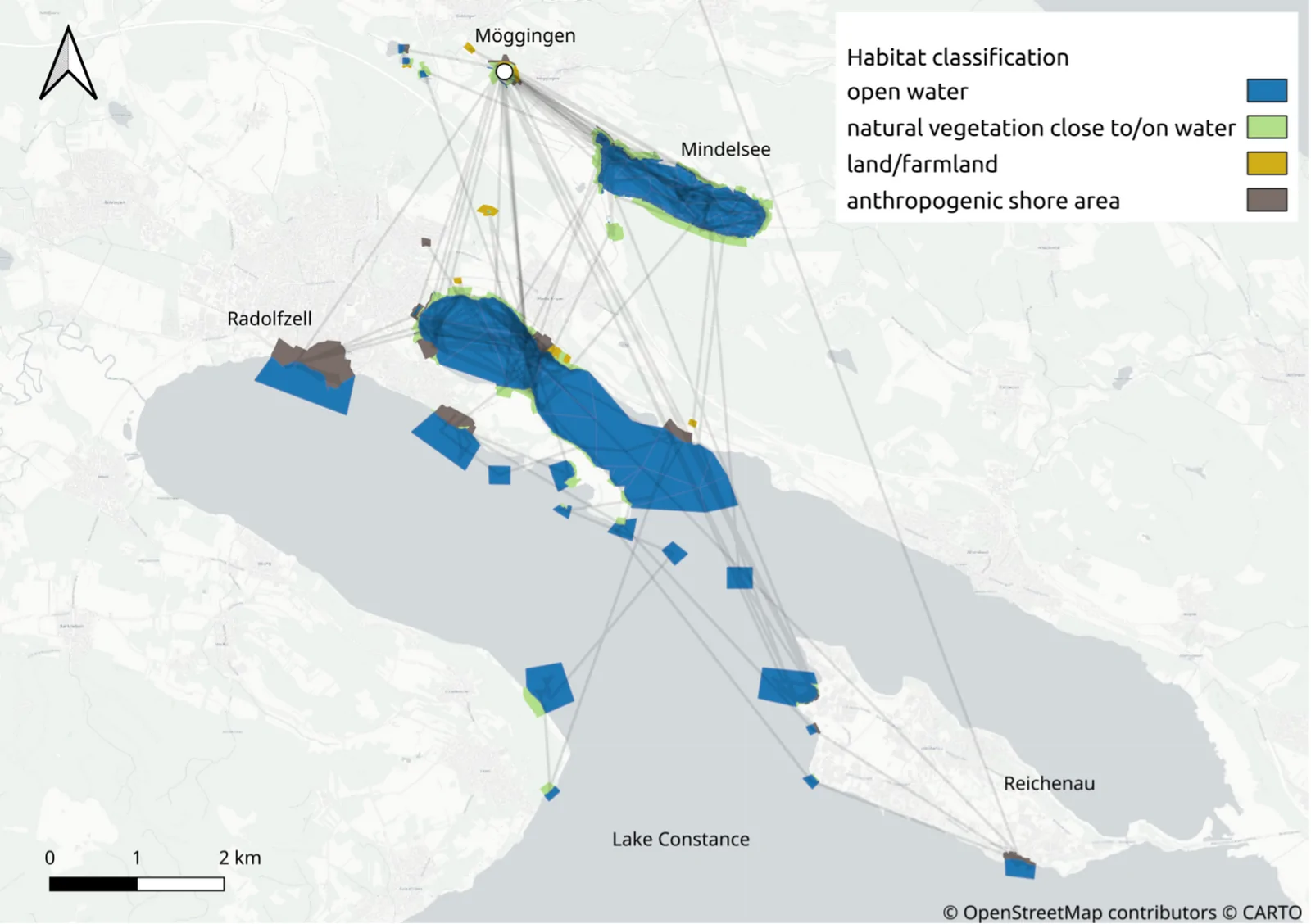
Habitat map and movement tracks of GPS-tagged mallards in the main study area in the Lake Constance region, Germany. The habitat map shows the four land cover classes used in the analyses, displayed on a Carto Light basemap. Light grey lines represent the movement tracks of all tracked individuals that remained within the main study area during the study period (all individuals except one, shown separately in Fig. S8). The white circle indicates the capture and release site. A detailed view illustrating the spatial resolution of the habitat classification is provided in Fig. S6-S7. Map background © OpenStreetMap contributors, Carto

To enable detailed classification of habitats in the area used by the ducks we took aerial photos of the area used by the mallards one week post release using a Cessna 172 airplane (Fig. S5). The photos were taken at 4500 ft (~ 1370 m) height with a EOS 7D Mark II camera with a focal length of 15 mm, which resulted in a orthomosaic with resolution of 25–30 cm/pixel. To generate a land cover map, we manually drew polygons to represent different land cover types in QGIS v. 3.10.4 software [58]. One individual left the area covered by the aerial photos within the duration of the study. The habitat types covering the locations recorded outside the core area were classified using Google Maps Satellite Imagery from within QGIS. Habitats were classified as belonging to four categories; (1) open water, (2) (semi)natural vegetation close to/on water, (3) anthropogenic shore area and (4) land/farmland (Fig. 2 and S6-S7 and Table S2). “Open water” consisted mainly of lakes, but also included a small number of artificial water bodies and streams. “(Semi)natural vegetation close to/on water” contained different types of habitats that may provide shelter for mallards close to-, or on water (marsh, forested marsh, reed bed, trees hanging over the water, logs in water, large patches of pond lilies, and forest in close proximity to open water). “Anthropogenic shore area” were areas used by humans, such as populated lakeside (i.e. swimming/recreational areas) or urban areas. “Land/farmland” consisted of agricultural areas such as farmland and grassland. Finally, each GPS fix was assigned a habitat classification by intersecting the birds’ locations with the land cover map in QGIS. Because habitats were classified based on locations used by the mallards, the resulting distribution of habitat categories reflects habitat use rather than habitat availability and was not expected to be evenly distributed among categories. To minimize bias due to personal interpretation, two researchers (R.T.K. and E.J.) conducted the habitat classification individually and the habitat use analysis was run for each classification. All results shown and discussed in the main manuscript are from the model ran with information from the land cover map from R.T.K., whereas the results from the model ran with information from the land cover map from E.J. are shown in the supplementary files. Sex was not included as a fixed effect in this model because habitat use was summarized at the individual-day level, resulting in limited information to reliably account for sex when estimating treatment effects. Females were evenly distributed between treatments (one control and one LPS individual), reducing the potential for sex to confound treatment comparisons.

To estimate and compare the proportional use of the four habitat types in the two treatment groups at different time points after stimulation, we fitted a multinomial logistic regression model allowing for a multinomial response variable (habitat types; Ntzoufras 2011 [59]). We included the number of GPS fixes in each habitat type per day and individual as the multinomial response variable, and combined the two factors treatment and days after stimulation (treatment: LPS or control, time point: 1–7 days post stimulation) and included this as the explanatory variable. To account for the non-independence of observations from the same individual, we included individual as a random factor.

The parameters of the multinomial logistic regression model were estimated in a Bayesian framework using Markov chain Monte Carlo simulations. For this purpose we ran WinBUGS v. 1.4 [60] in R using the R2WinBUGS v. 2.1–22 R package [61]. We ran three Markov chains with 500,000 iterations and a burn in period of 50,000. The chains were thinned by ten (nine out of ten observation were discarded) to reduce autocorrelation. We assessed the convergence graphically and by the R-hat value, which were all close to one (Table S3-S4) [62]. We extracted the means as estimates and the 2.5% and 97.5% quantiles to describe the 95% credible intervals (95% CrI [56]) from the Markov chains. For pairwise comparison of habitat use between the treatment groups, we calculated the posterior probability (certainty) of the hypothesis that the mean proportional use of a particular habitat type in one group is larger than the mean proportional use of a particular habitat type of the other group. We considered an effect to be substantial if the certainty of being larger was > 0.95.

## Results

### Movement patterns in the wild mallards

#### Overall movement in the control and treatment group

Most mallards moved between the pond where they were initially captured in Möggingen, and the shores of Lake Constance or smaller lakes in the vicinity of the capture location (Fig. 2 and S8). All individuals remained within 16 km of the capture site during the period for which GPS data was recorded during the study period (seven days). The two furthest step lengths recorded (with a time interval of approximately 20 min) were 13.4 and 9.6 km. Both these distances represent a single flight of one control individual from Reichenau Island to a small stream next to a trout farm in the vicinity of Eigeltingen three days after release (Fig. S8). The furthest step length in a LPS treated individual was 4.1 km; this corresponds to a flight four days after stimulation from a boat jetty in Radolfzell at Lake Constance to the pond where the mallards were initially captured in Möggingen.

#### Effect of treatment on movement patterns and habitat use

To determine if the immuno-stimulant caused behavioural changes in the mallards, we assessed and compared the movement patterns and habitat use in the LPS immune challenged and control mallards for one week after release.

**Fig. 3:**
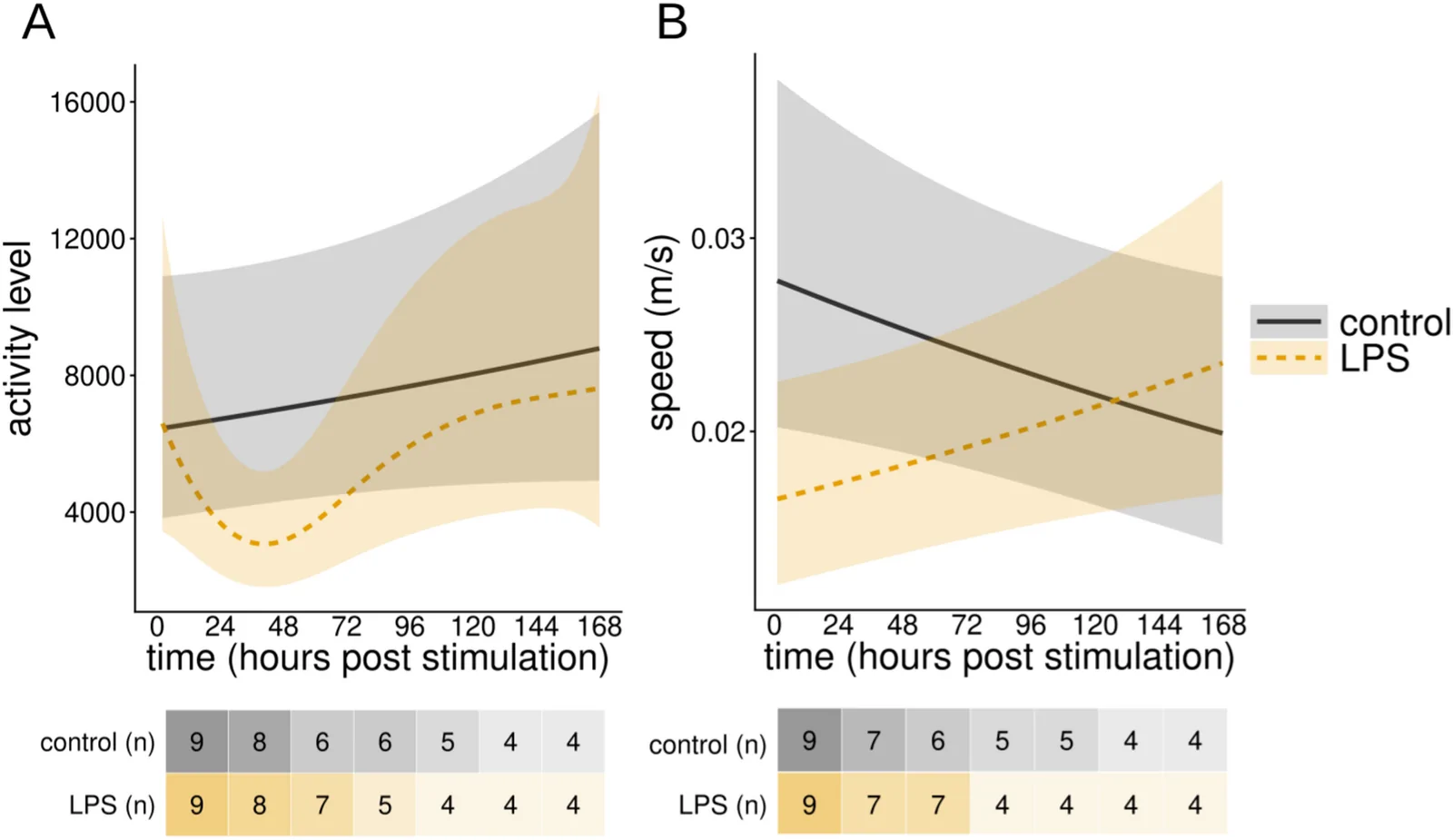
Movement patterns following challenge with LPS stimulants. Changes in (**A**) activity level, and (**B**) minimum speed (meter per second) measured remotely using bio-loggers. Model-predicted mean and 95% confidence interval for each treatment group is plotted until one week post stimulation. An activity value of 0 means no activity. The number of individuals with available logger data decreased from nine to four per treatment group over the seven-day study period (Fig. 1, S9 and S11). In total, 1726 data points containing information on activity level per hour (calculated from 25160 ACC bursts) were included in the analysis. In total, 4378 high quality GPS points remained for the speed analysis after filtering. Numbers beneath each plot indicate the number of tracked individuals with at least one data point available on each day

First, we compared activity levels measured using high definition accelerometers that record acceleration along three axes [63]. Across 1,785 observations from 18 individuals (Fig. 1 and S9), LPS-treated mallards showed an approximately 33% lower activity level than control mallards. Temporal dynamics were treatment-specific: the activity level remained relatively constant in the control individuals whereas the activity level in the LPS-treated individuals changed non-linearly following stimulation (Fig. 3A and Table S5). The model predicted lower general activity levels in the LPS-treated group than in the control group from 20.4 to 72.4 h post-stimulation (Fig. 3A and Dataset S1). During this period, the 95% confidence interval of the LPS treatment prediction did not include the estimated mean of the control prediction, indicating clear graphical separation between the predicted trajectories. Activity level also varied across day periods, with mallards showing the highest activity levels during the civil twilight evening and astronomical twilight morning, and the lowest activity levels during the early night (Fig. S10 and Table S5). Among-individual differences in baseline activity were modest (ICC ≈ 0.04), with most variability occurring within individuals. Activity levels showed moderate positive temporal autocorrelation (AR(1) φ = 0.25), indicating that individuals tended to show similar activity levels across consecutive measurements (Table S5).

Second, we calculated and compared the minimum speed and step length in free living mallards using GPS coordinates. Across 4,378 observations from 18 individuals (Fig. 1 and S11), LPS-treated mallards showed a ~ 20% decrease in movement speed (meters per second) compared to controls. Speed changed linearly over time in both treatment groups, but with opposite trends: in control mallards, speed decreased slightly over time, whereas in LPS-treated mallards, speed increased over the same period (Fig. 3B and Table S6). Model predictions indicated lower speed in the LPS-stimulated group than in the control group until 56.6 h post stimulation (Fig. 3B, and Dataset S2). Also for speed, we observed variation in the movement metric across the day period, with mallards travelling at higher speed during the civil twilight evening and astronomical twilight morning, and at lower speed during the early night (Table S6 and Fig. S12). Variation in baseline speed among individuals was small (ICC ≈ 0.007), with the majority of the variability occurring within individuals. Speed showed moderate temporal autocorrelation (AR(1) φ = 0.34), reflecting that an individuals speed tended to persist across consecutive measurements (Table S6). The results from the GAMM comparing the minimum distance between consecutive locations (step length) closely matched the patterns for speed (Table S7). Specifically, model predictions indicated that step lengths were shorter in the stimulated group than in the control group until 58.3 h post-stimulation (Fig. S13-S15, Table S7 and Dataset S3). Including sex as an additional fixed effect produced comparable treatment patterns and did not alter the interpretation of the effects observed for activity level, speed, or step length (Supplementary Tables S8–S10; Fig. S16–S17).

**Fig. 4:**
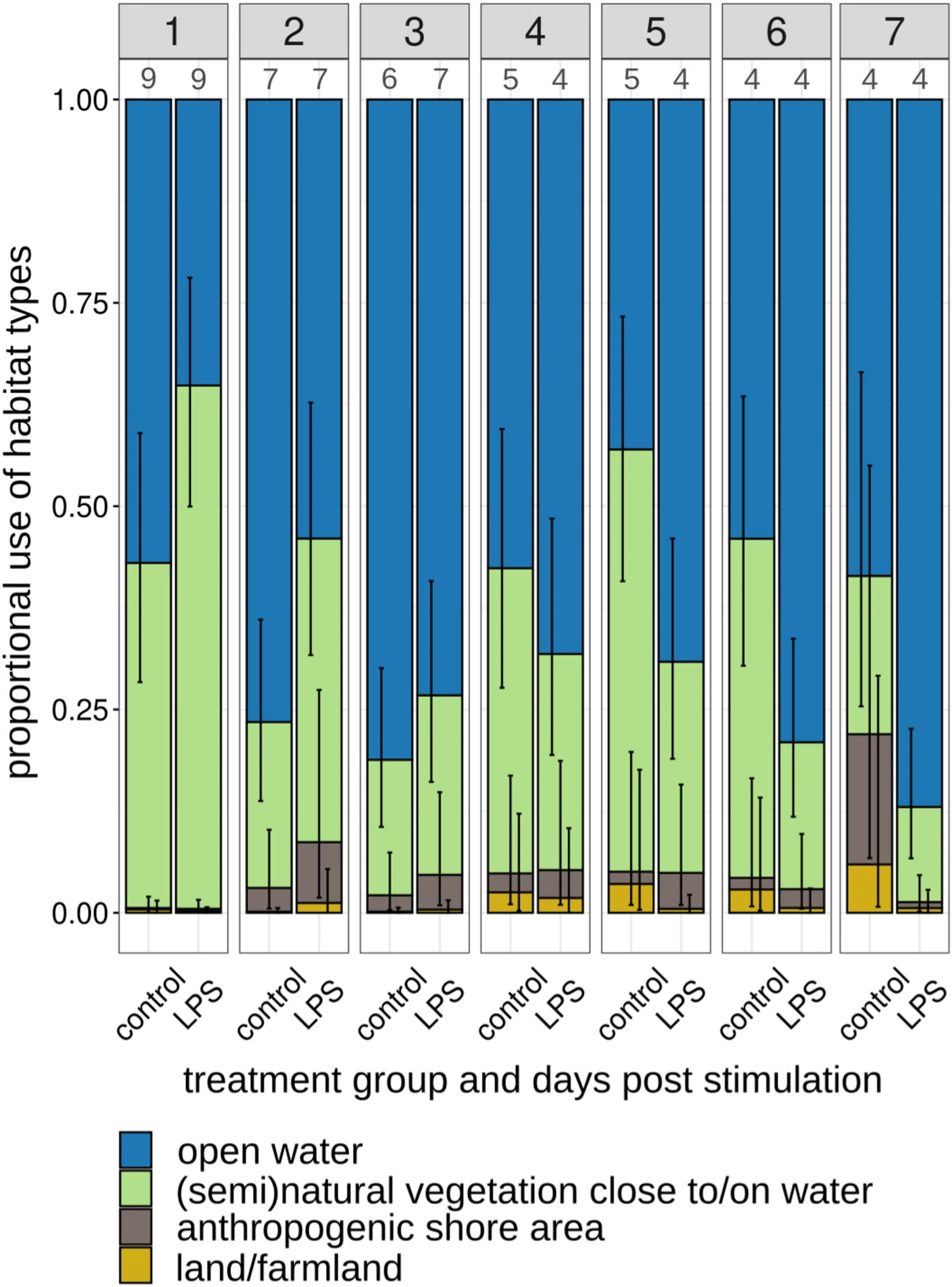
Habitat use following challenge with the immune-stimulant LPS. Cumulative mean proportion of the habitat types used by the mallards and cumulative 95% credible interval as estimated from the posterior distribution of the multinomial model. Habitat categories are shown in a consistent order from top to bottom as open water, (semi-)natural vegetation close to/on water, anthropogenic shore area, and land/farmland. The number of individuals included in the habitat use analysis decreased from 9 to 4 in each treatment group during the 7 days which the study took place (Fig. 1 and S11). In total, habitat type information for 4394 GPS fixes (HDOP ≤ 2) was included in the analysis. The measured habitat use proportion is shown in figure S15. Grey numbers above each bar indicate the number of tracked individuals with data available per day and treatment group

Third, to evaluate whether immune challenged mallards use their environment differently from healthy individuals, we assessed and compared the proportional use of four classes of habitat types each day after release in each group for a total of seven days (Fig. S18). We identified an effect of the LPS treatment on habitat use (Fig. 4, Table S3 and S11), with immune challenged individuals spending more time in (semi)natural vegetation close to or on water (certainty > 97%) and less time on open water (certainty > 95%) than the control group during the first two days after treatment. From day five to seven, this pattern was reversed, with immune-challenged individuals spending more time on open water (certainty > 98%), and from day five to six, less time in (semi)natural vegetation close to or on water (certainty > 98%), than the control group. In addition, they spent less time in anthropogenic shore areas on the seventh day (certainty > 99%) and land/farmland on the fifth and seventh day after treatment (certainty > 96%) than the control group (Table S11). Habitat use analysis was conducted twice using the independently produced classification maps with statistically identical results (Tables S3-S4 and S11–S12, Fig. 4 and S19).

## Discussion

Sickness behaviour represents an important link between immune responses and animal ecology, as immune-mediated changes in behaviour can influence how individuals move and interact with their environment. Although behavioural responses to experimental immune activation have been investigated in free-ranging songbirds [22–26], comparable studies in waterfowl remain scarce. Addressing this gap is important for understanding how sickness behaviour may shape the ecology of waterfowl and is particularly relevant because waterfowl are important hosts of pathogens of veterinary and public health concern. This relevance is illustrated by recent work using large-scale telemetry datasets, which found an association between waterfowl movement and the spatial distribution of highly pathogenic avian influenza in wild waterfowl [64]. Here, we found that immune-challenged mallards travelled shorter distances, moved at lower speeds, showed lower activity, and used different habitats than control individuals during the acute phase response. These findings demonstrate that acute immune activation can alter both movement and fine-scale habitat use in free-ranging mallards and highlight the value of high-resolution biologging for quantifying sickness behaviour in wildlife.

Our acceleration data showed that mallards substantially reduced their activity level 2–3 days after PLGA-microsphere-mediated LPS injection. These results are similar to those by Lennon et al. [19], showing that free-living blackbirds reduce their daily activity following experimental LPS stimulation. In contrast, a previous study in captive Pekin ducks found no alteration in activity following LPS treatment when measured using accelerometers [15]. In our previous study, LPS-treated and control mallards in captivity likewise did not differ in activity levels when assessed using accelerometer data, despite evidence of a systemic immune response to the treatment [36]. Similar contrasts between captive and free-ranging conditions have been observed in chicken, where free-roaming individuals showed a greater reduction in movement than caged individuals after injection with LPS [14]. Together, these findings raise the possibility that the expression of sickness behaviour depends partly on environmental context. Free-ranging mallards need to move between resources, respond to environmental conditions, and balance movement against risks such as predation, potentially creating behavioural consequences of immune activation that are less apparent under captive conditions.

To our knowledge, only one study has used accelerometers to investigate sickness behaviour in naturally infected wild mallards. Bengtsson et al. [27] found no difference in overall dynamic body acceleration (ODBA) between LPAIV-infected and uninfected mallards when averaged over one week. This contrast with our findings should be interpreted cautiously because LPS stimulation and natural LPAIV infection induce different host responses. Mallards are natural reservoirs of LPAIV and often show few clinical signs following infection, despite the multifaceted host response elicited by viral infection [24, 65–67]. In contrast, LPS induces a measurable systemic acute-phase response in this species [28, 56, 57], including changes in body temperature, heart rate, leukocyte composition, and whole-blood gene expression [36]. The contrasting findings may therefore reflect differences in the nature and magnitude of the responses elicited by LPS and LPAIV, as well as differences in study design. Experimental approaches therefore complement studies of naturally infected individuals by allowing specific aspects of immune activation and sickness behaviour to be investigated under controlled conditions, while studies of naturally infected individuals remain essential for understanding behavioural responses in the context of natural host–pathogen interactions [68].

The microsphere formulation was used to extend the duration of the LPS-induced response beyond that observed following conventional LPS administration, providing a longer window to observe sickness-related behavioural responses while reducing the relative influence of capture and handling. In our previous study, the increase in body temperature in mallards following conventional LPS administration lasted for less than one day [36], whereas the pilot experiment conducted as part of the present study using the microsphere formulation showed elevated body temperature for approximately 3 days. This duration overlaps temporally with the period over which immune and antiviral responses have been reported following natural infections in ducks [35, 69, 70]. However, this temporal overlap does not imply that the magnitude or dynamics of the LPS-induced response are comparable to those occurring during infection, as LPS-induced immune activation does not involve the pathogen-specific interactions and processes associated with natural infection.

In addition to enabling the detailed study of sickness behaviour, biologging sensors have shown promise for detecting illness in domestic, managed and free-ranging animals [71–75]. Behavioural changes associated with bovine ephemeral fever [72], bovine respiratory disease [73], and African swine fever [74] have been detected using accelerometer data, while GPS-derived movement trajectories have also been used to identify changes associated with disease progression [75]. More broadly, movement and tracking data are increasingly being incorporated into approaches for monitoring and forecasting disease dynamics [76]. Our results add to this growing body of work by demonstrating that accelerometer- and GPS-derived behavioural metrics can reveal sickness-related behavioural changes in free-ranging mallards. Experimental studies such as ours, together with previous experimental work [22–26], can thus help identify behavioural changes that may be relevant indicators of infection. However, before such behavioural responses can be used to inform disease surveillance, they need to be evaluated in naturally infected individuals across a range of pathogens to determine whether they reliably distinguish infected from non-infected individuals and to establish appropriate thresholds for identifying affected animals.

The GPS data complement the accelerometry results by showing how reduced activity during the acute phase response translated into changes in movement across the landscape. By calculating and comparing movement speed and step length between immune-challenged and control birds, we found that wild mallards moved at lower speed and over shorter distances during the LPS-induced acute phase response. Similar reductions in movement have been reported in migrating passerines following experimental LPS stimulation, where immune-challenged birds travelled shorter distances during autumn stopover [20]. Likewise, weak negative associations between LPAIV infection and daily cumulative distance travelled in displacements exceeding 100 m have previously been reported in free-living adult male mallards [29]. The observed decrease in speed and step length in our study may reflect a reallocation of energy from movement towards immune responses, including fever, or a reduced motivation to travel further distances in search of food.

In addition to altering movement, acute immune activation was associated with changes in habitat use in free-ranging mallards. Our results show that mallards spent more time in sheltered areas (natural vegetation close to or on water) and less time in open water during the peak of the acute phase response. Towards the end of the study, however, the model predicted a potential shift in the opposite direction, with immune-challenged mallards spending more time in open water and less time in sheltered areas than control birds. These findings extend previous work on sickness behaviour by showing that immune-mediated responses influence not only activity and movement, but also fine-scale habitat use in free-ranging mallards. The increased use of sheltered habitats during the acute phase response may reflect an increased need for rest, predator avoidance, and/or reduced motivation or ability to seek food and water [1, 2, 57]. Reduced foraging motivation is consistent with previous work showing that Pekin ducks injected with LPS reduce their food consumption and lose body mass during the febrile response [15]. The increased use of open water habitats at the end of our study may in turn reflect compensatory increases in foraging following the acute phase response. However, fewer individuals contributed data towards the end of the study, resulting in greater uncertainty around these later predictions. Other habitat-use differences detected during this period were also limited to individual days. We therefore interpret the late-study habitat-use patterns cautiously and do not draw strong conclusions about behavioural changes during recovery.

In contrast to large-scale movements, which are known to influence pathogen spread in migratory systems [77, 78], comparatively little is known about how sickness-related changes in local movement and habitat use may influence host interactions and pathogen transmission at smaller spatial scales. Reductions in local movement could alter the spatial distribution of infected individuals and their interactions with susceptible individuals, while shifts in habitat use could alter where infected individuals spend time and how they use shared environments. For example, if naturally infected individuals show similar increases in sheltered habitat use as LPS-treated individuals in our study, this could influence where transmission opportunities arise. For pathogens transmitted via environmentally persistent infectious particles, such changes in habitat use could influence local exposure patterns, although the extent to which this occurs remains largely unknown, including for AIV [79, 80]. Experimental work in mallards has shown that environmental conditions, including exposure route and viral concentration in water, can influence infection dynamics, including infection onset, duration, and shedding patterns [81], highlighting the potential importance of local habitat conditions for transmission. Our results do not provide evidence that sickness-related habitat use affects transmission; rather, they identify a behavioural mechanism that could be investigated in future studies combining behavioural monitoring with virological surveillance, environmental sampling, and contact network modelling.

Because immune responses can differ between sexes [82–84], and male and female mallards can differ in behaviour [85], sickness behaviour may also differ between sexes. Our study was initially designed to include a more balanced representation of the sexes; however, fewer birds than anticipated were captured during the field season, and the majority were males. We initially tagged four females, two in each treatment, but one logger from each treatment failed to transmit any data. Consequently, only one female per treatment contributed data to the final dataset, which was therefore male-biased and limited our ability to assess potential sex-specific behavioural responses. As a sensitivity analysis, we included sex as an additional fixed effect in the movement models, which resulted in virtually unchanged estimated treatment effects. Nevertheless, larger and more balanced samples would be needed to formally evaluate whether responses to immune stimulation differ between males and females.

Technical issues with the biologgers resulted in progressively fewer individuals contributing data over the study period. In several loggers, declining battery levels were associated with less frequent location recording and, eventually, no data transmission. Together, the absence of stationary location data indicative of mortality, similar patterns of data transmission between treatment groups, and the successful recapture of a healthy bird with a non-transmitting logger suggest that data loss was primarily technical rather than treatment-related. We included all observations from functioning biologgers in the analyses. The mixed-model framework accommodates the resulting unbalanced sampling structure, allowing individuals to contribute different numbers of observations over the study period. As the number of contributing birds decreased over time, uncertainty intervals around model estimates generally became wider at later time points. Importantly, the primary behavioural differences between treatment groups occurred during the first days of the experiment, when sample sizes were largest, consistent with the prolonged elevation in body temperature observed over this period in the pilot study following administration of microsphere-encapsulated LPS.

Our study highlights several directions for future research on sickness behaviour in wildlife. Experimental immune challenges during migration could reveal whether sickness responses alter migratory movement or stopover behaviour. More detailed studies combining behavioural monitoring with proximity logging [86, 87], virological surveillance, and habitat-specific environmental sampling could help determine how sickness-related changes in movement and habitat use influence interactions and processes relevant to pathogen transmission. This may be particularly relevant in landscapes where wild waterbirds move between larger waterbird aggregation sites and surrounding agricultural areas, potentially linking locations where wild birds congregate with areas where domestic poultry are kept. Finally, comparisons between experimentally challenged and naturally infected individuals will be important for determining whether and how behavioural responses vary across different host–pathogen contexts. Such comparisons could also help determine whether behavioural changes observed following experimental immune activation are detectable during natural infections and could eventually contribute to disease surveillance.

## Conclusion

This study provides evidence that experimental immune activation induces sickness-related behavioural changes in free-ranging mallards. Using high-resolution GPS, accelerometer data, and habitat mapping, we show that LPS challenge reduces movement and activity and alters habitat use during the acute phase response. These findings highlight the value of high-resolution biologging for quantifying sickness-related changes in behaviour in free-ranging wildlife and provide a basis for future studies investigating how such changes may influence animal ecology and ecological interactions, including their potential relevance to pathogen transmission under natural host–pathogen contexts.

## Supplementary Information

Below is the link to the electronic supplementary material.

Supplementary Material 1

## Data Availability

The GPS and acceleration data has been uploaded to the Movebank Data Repository https://doi.org/10.5441/001/1.721 [88]. All other datasets and the R code used for the analyses are available on Figshare at https://doi.org/10.6084/m9.figshare.30083755 [89].

## Abbreviations

ACC: Acceleration
AIV: Avian influenza virus
CI: Confidence intervals
CrI: Credible Intervals
FDA: Food and Drug Administration
GAMM: Generalized Additive Mixed Model
GPS: Global Positioning System
GSM: Global System for Mobile Communications
HDOP: Horizontal dilution of precision
LPAIV: Low pathogenic avian influenza virus
LPS: Lipopolysaccharide
ODBA: Overall dynamic body acceleration
PBS: Phosphate-buffered saline
PIT: Passive Integrated Transponder
PLGA: Poly(lactic-co-glycolic acid)
poly I.C: Polyinosinic: polycytidylic acid
