# Supplementary material for: Immune-challenged wild mallards move less and spend more time in sheltered habitats
Source: Mov Ecol. 2026 Oct 22;14:76. doi: 10.1186/s40462-026-00715-0 (PMC13700185; doi:10.1186/s40462-026-00715-0)
Supplement: Supplementary file 1 [file 40462_2026_715_MOESM1_ESM.pdf]

Supplementary files

# Immune-challenged wild mallards move less and spend more time in sheltered habitats

Elinor Jax[1,2]
Inge Mueller[1,3]
Dennis Horvath[3]
Julia Koerner[3]
Wolfgang Fiedler[1,3]
Daniel Zuñiga[1,3]
Elena Werner[1]
Roman T. Kellenberger[4]
L. Michael, Romero [5]
Marcus Groettrup[3,6] †
Martin Wikelski[1,3]
Robert H. S. Kraus[1,3]

† Deceased

[1]Department of Migration, Max Planck Institute of Animal Behavior, Radolfzell, Germany
[2]Centre for Ecology and Evolution in Microbial Model Systems, Linnaeus University, Kalmar, Sweden
[3]Department of Biology, University of Konstanz, Konstanz, Germany.
[4]Department of Plant Sciences, University of Cambridge, United Kingdom
[5]Department of Biology, Tufts University, Medford, MA, United States of America
[6]Institute of Cell Biology and Immunology Thurgau (BITG) at the University of Konstanz, 8280 Kreuzlingen, Switzerland.

# Supplementary figures

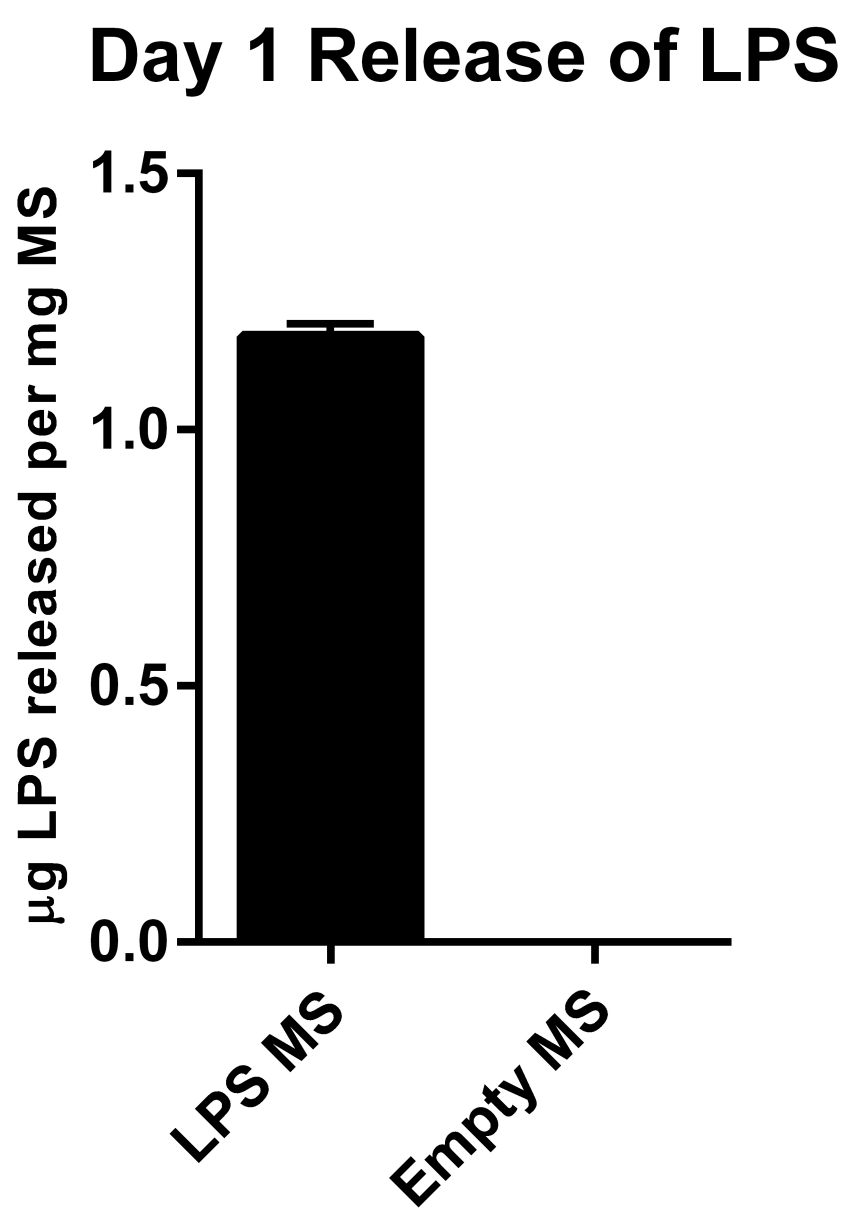


**Figure S1.** ***In vitro*** **release of LPS from PLGA-microspheres.** Released LPS was detected using a LAL chromogenic endotoxin test, with empty PLGA-microspheres as a negative control.

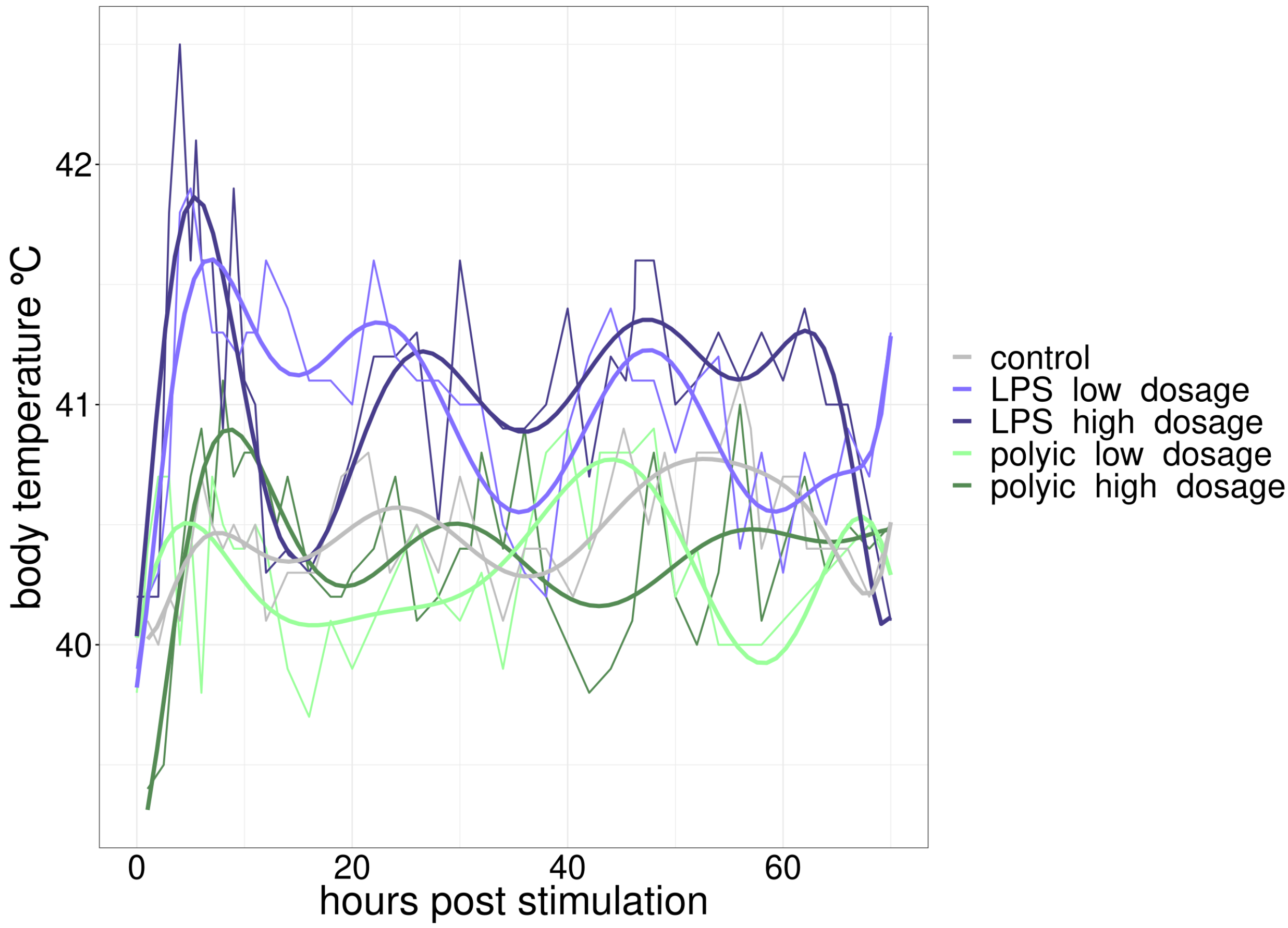


**Figure S2. Changes in body temperature in five individuals following challenge with microsphere embedded immune stimulants of different dosages.** Thin lines show the raw data, and the thick lines the smoothed conditional means estimated using the stat_smooth() function and the lm smoothing method from the ggplot2 R package, until 72 hours post stimulation.

LPS low dosage = 300 ug LPS/kg bodyweight (60 mg MS diluted in 3mL PBS)

LPS high dosage = 900 ug LPS/kg (180 mg MS diluted in 6mL PBS)

poly I:C low dosage = 3mg poly I:C/kg (60 mg MS diluted in 3mL PBS)

poly I:C high dosage = 9mg poly I:C/kg (180 mg MS diluted in 6mL PBS)

control = 180 mg empty microspheres diluted in 6mL PBS

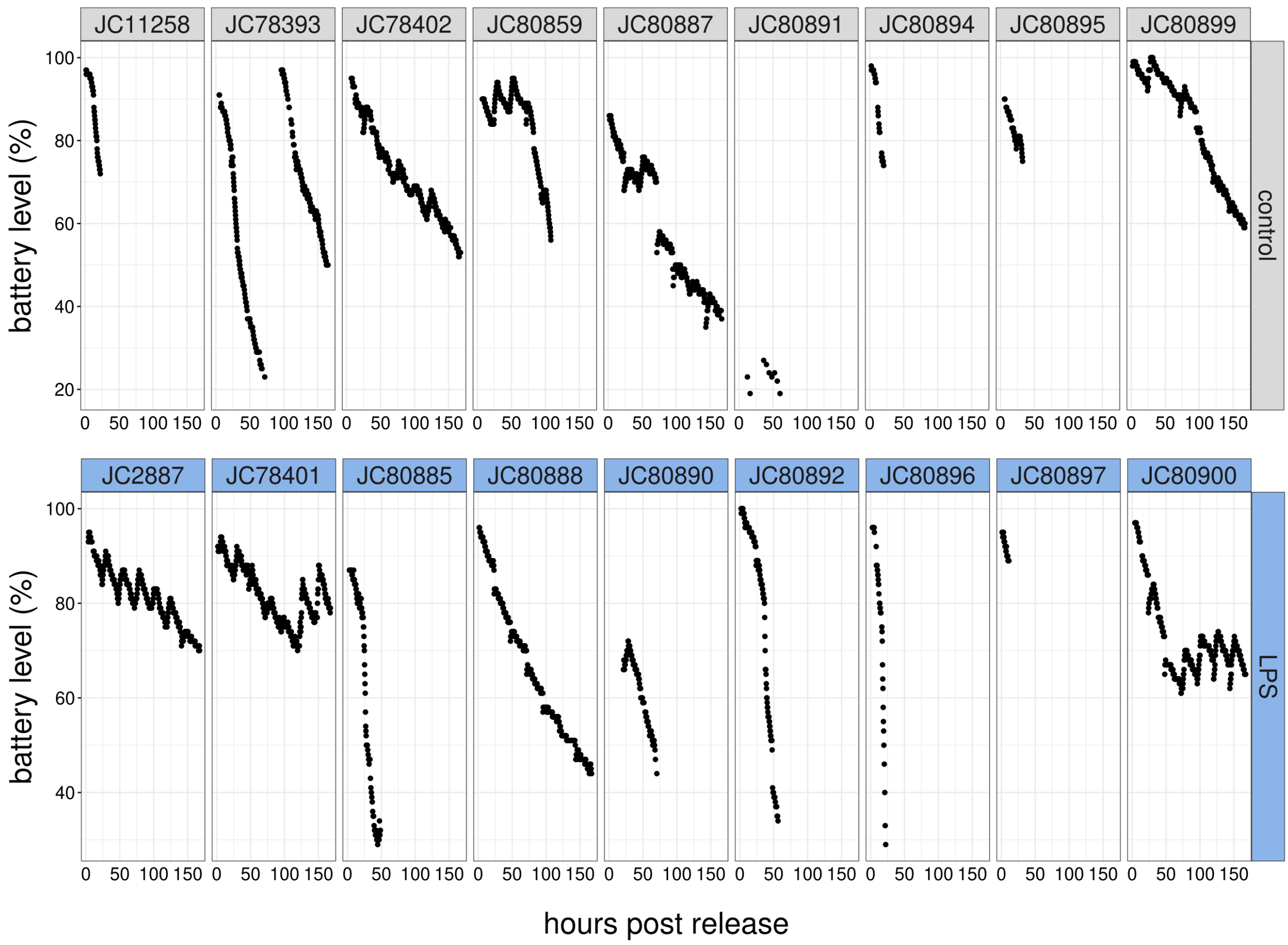


**Figure S3. Battery level of biologgers for each recorded GPS fix (HDOP≤2) one week post release.** Individual JC78393 was recaptured during the study period whereas its initial biologger was exchanged for new fully changed one.

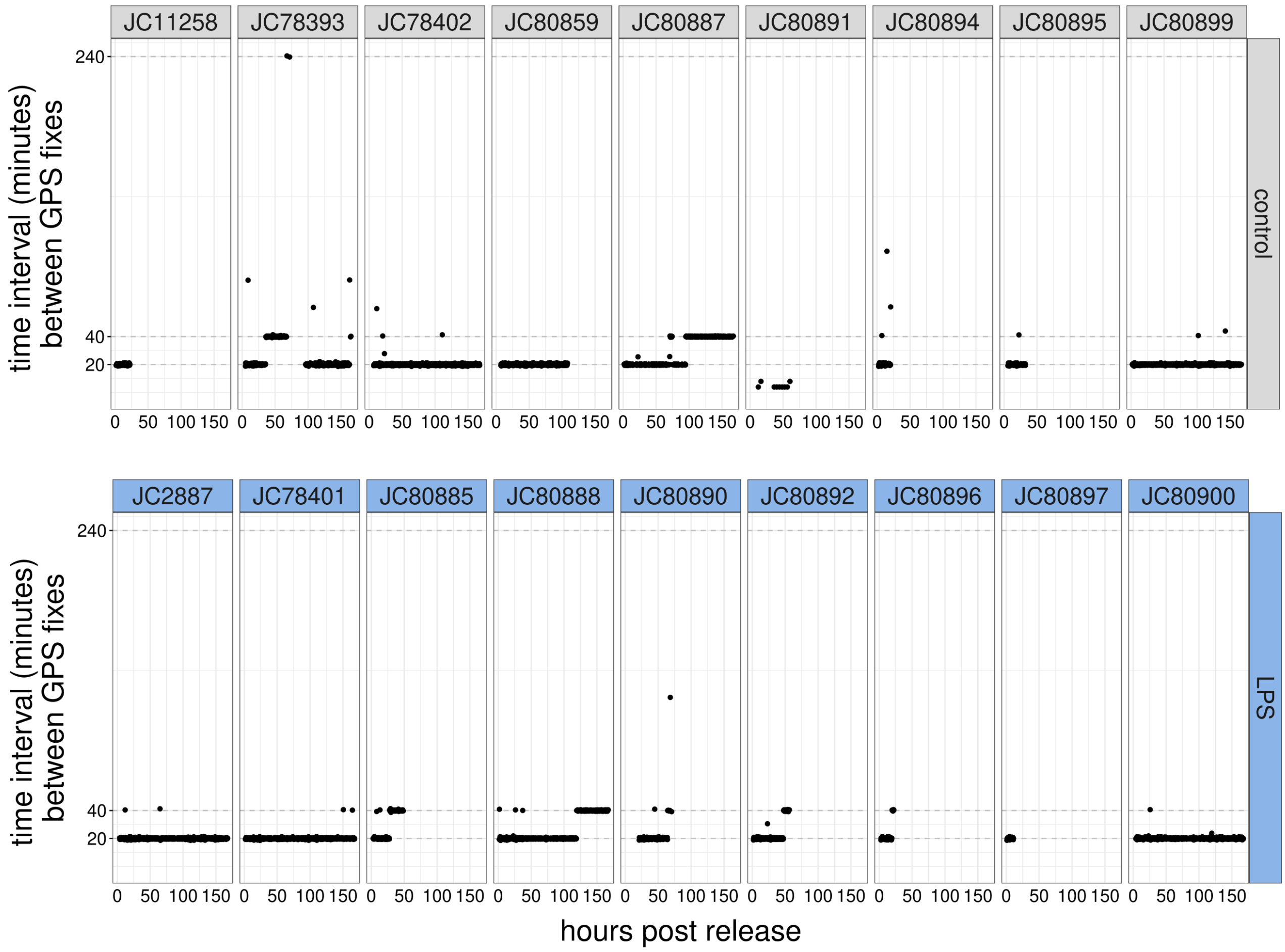


**Figure S4. Time interval between consecutive GPS fixes (HDOP≤2) one week post release.** Individual JC78393 was recaptured during the study period whereas its initial biologger was exchanged for new fully changed one.

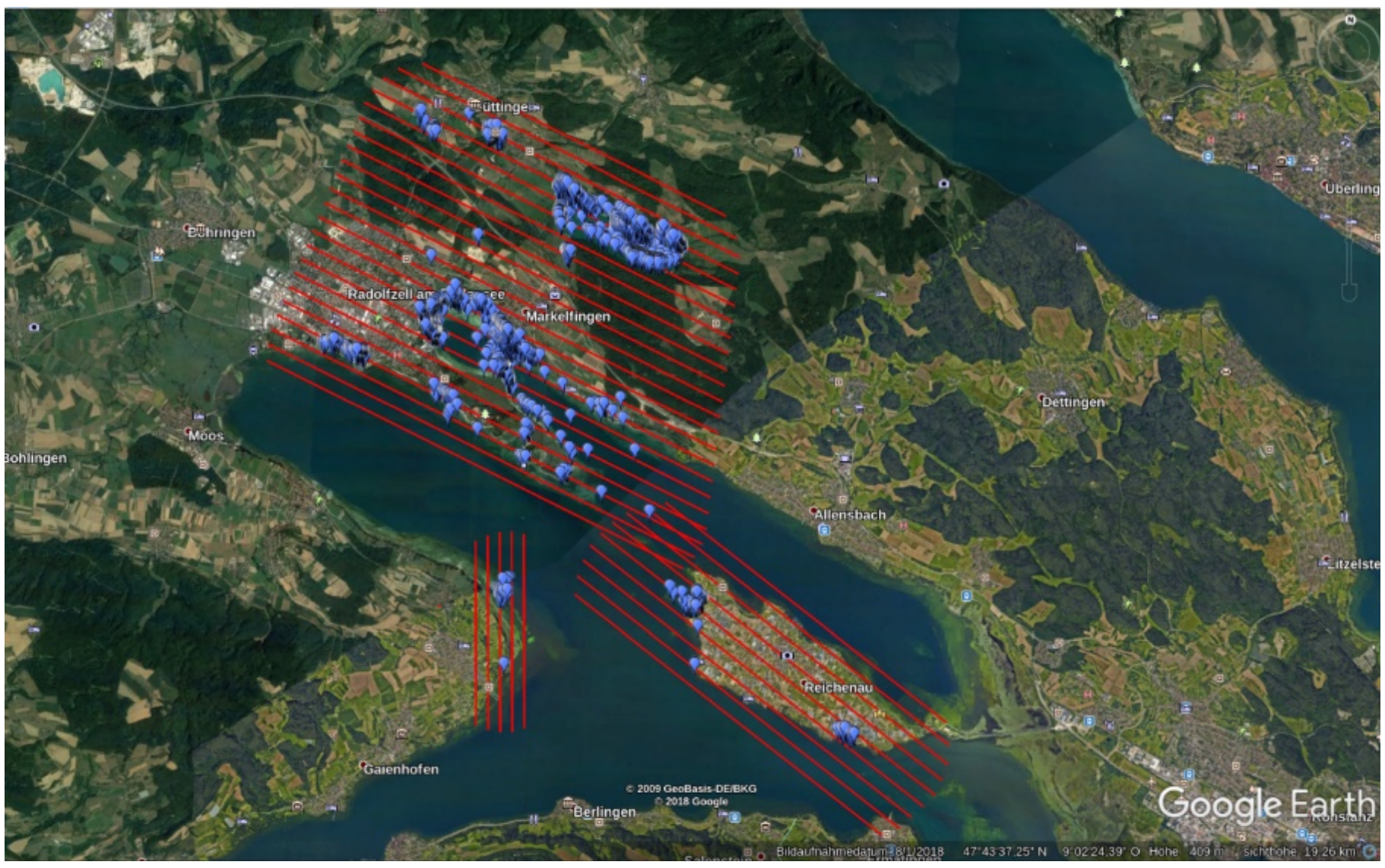

**Figure S5. Map showing GPS locations (blue dots) and the area covered by air plane.** Satellite image of the study area from Google Earth. (2019).

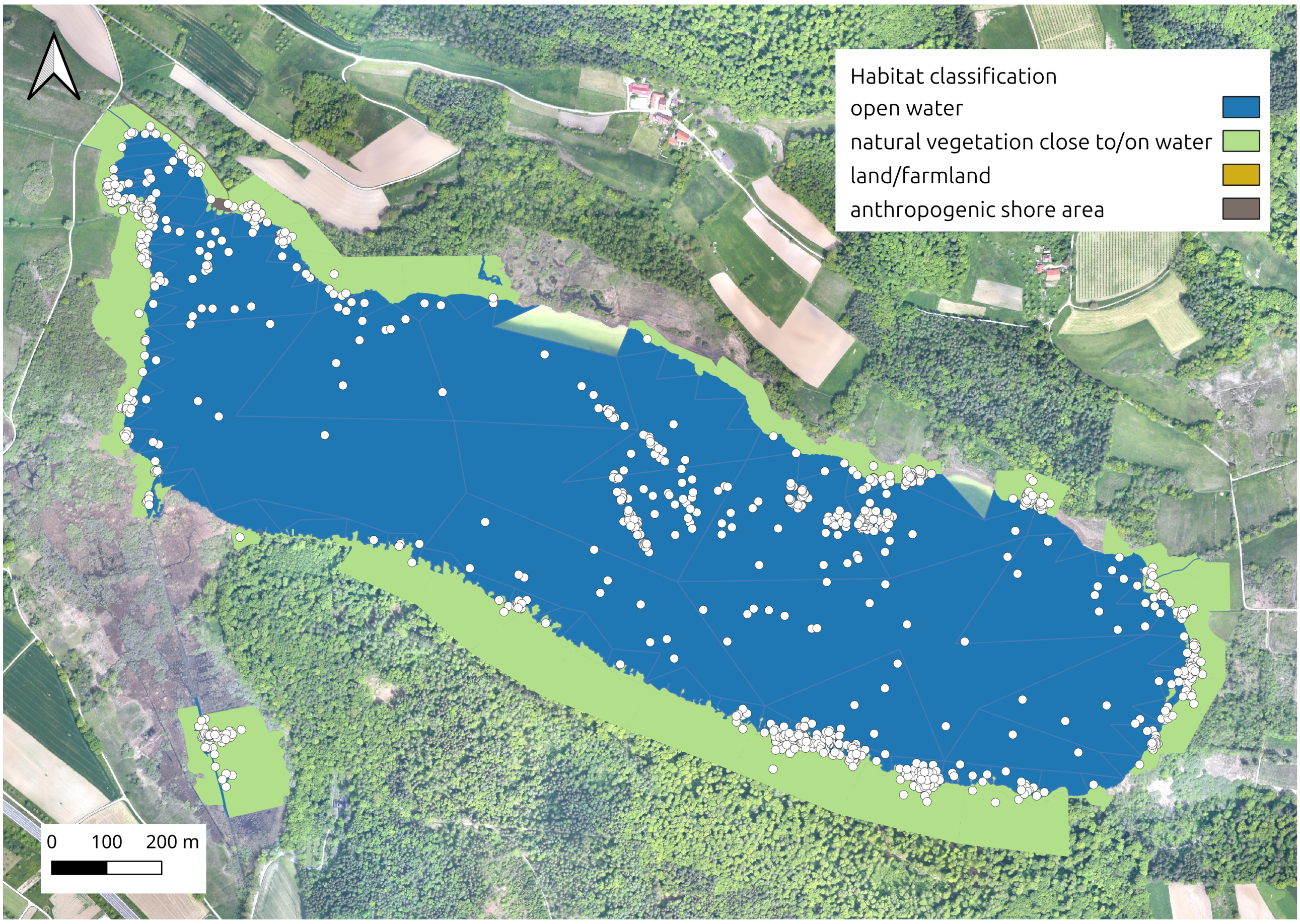


**Figure S6. Land cover map visualised on aerial photos for one of the lakes commonly visited by the mallards in the study area, the Mindelsee.** The white dots show recorded GPS locations of different mallards observed at this particular lake during the study. The legend shows the four habitat types used by the mallards across the whole study area (the land cover map generated for this part of the study area contains three of the four habitats).

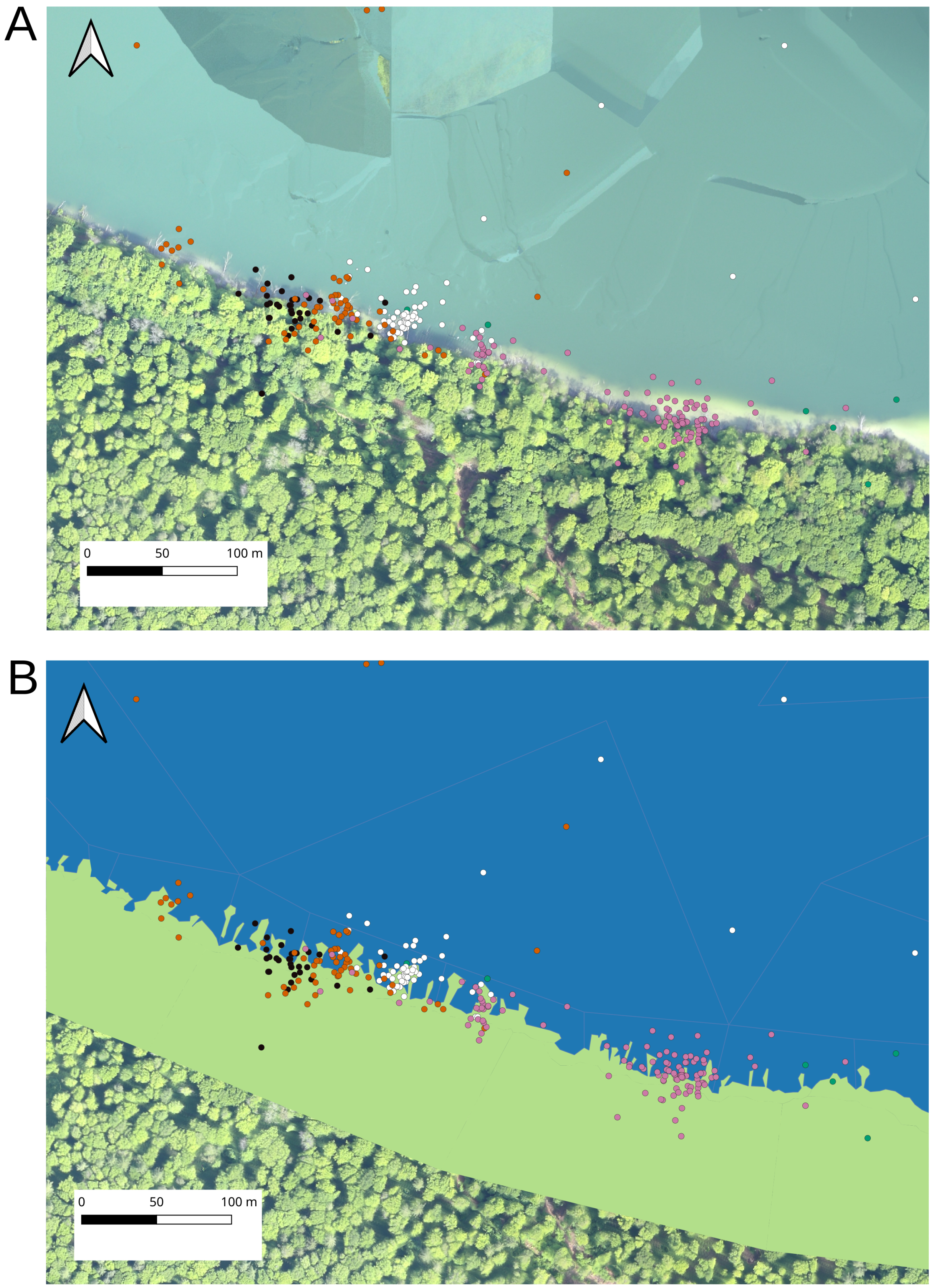


**Figure S7. Land cover map visualised on aerial photos - showing a close-up of the habitat classification along the shoreline of Lake Mindelsee.** Overhanging trees and/or logs in the water, as well as forest in close proximity to open water were classified as "(Semi)natural vegetation close to/on water" whereas a uncovered water surfaces were classified as "Open water". High quality GPS fixes recorded from mallards are visualised on the map, each colour represents one individual mallard. Once areas to be classified had been identified, dots representing GPS fixes from mallards were removed to allow for detailed classification without knowledge of exact positions of the birds.

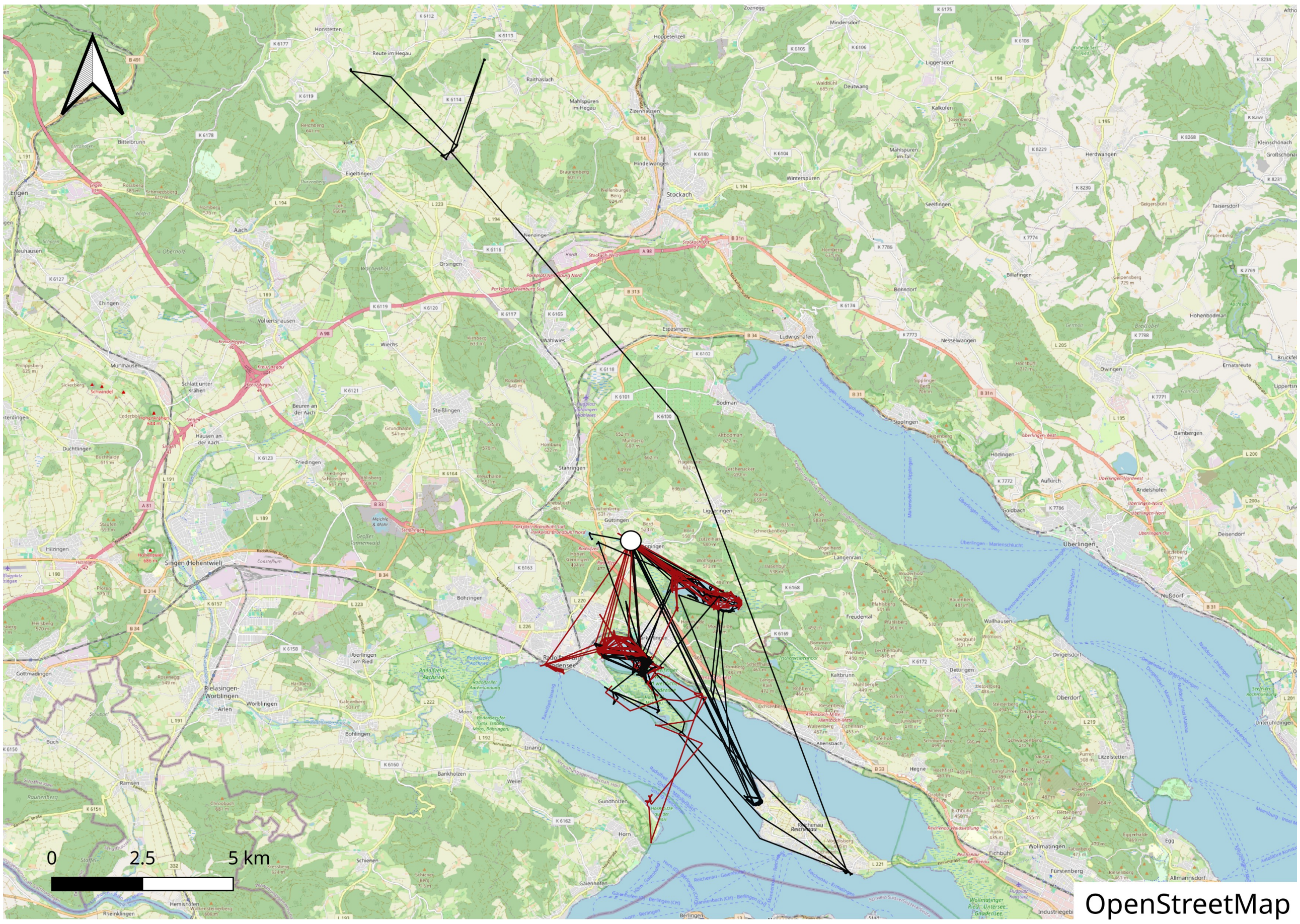


**Figure S8. Tracks showing movements of the mallards one week post release in the Lake Constance area, Germany.** Tracks from control birds are shown in black (n=9) and from LPS challenged birds in red (n=9). The site of capture and release is shown as white circle. Map data from OpenStreetMap 2026-02-09

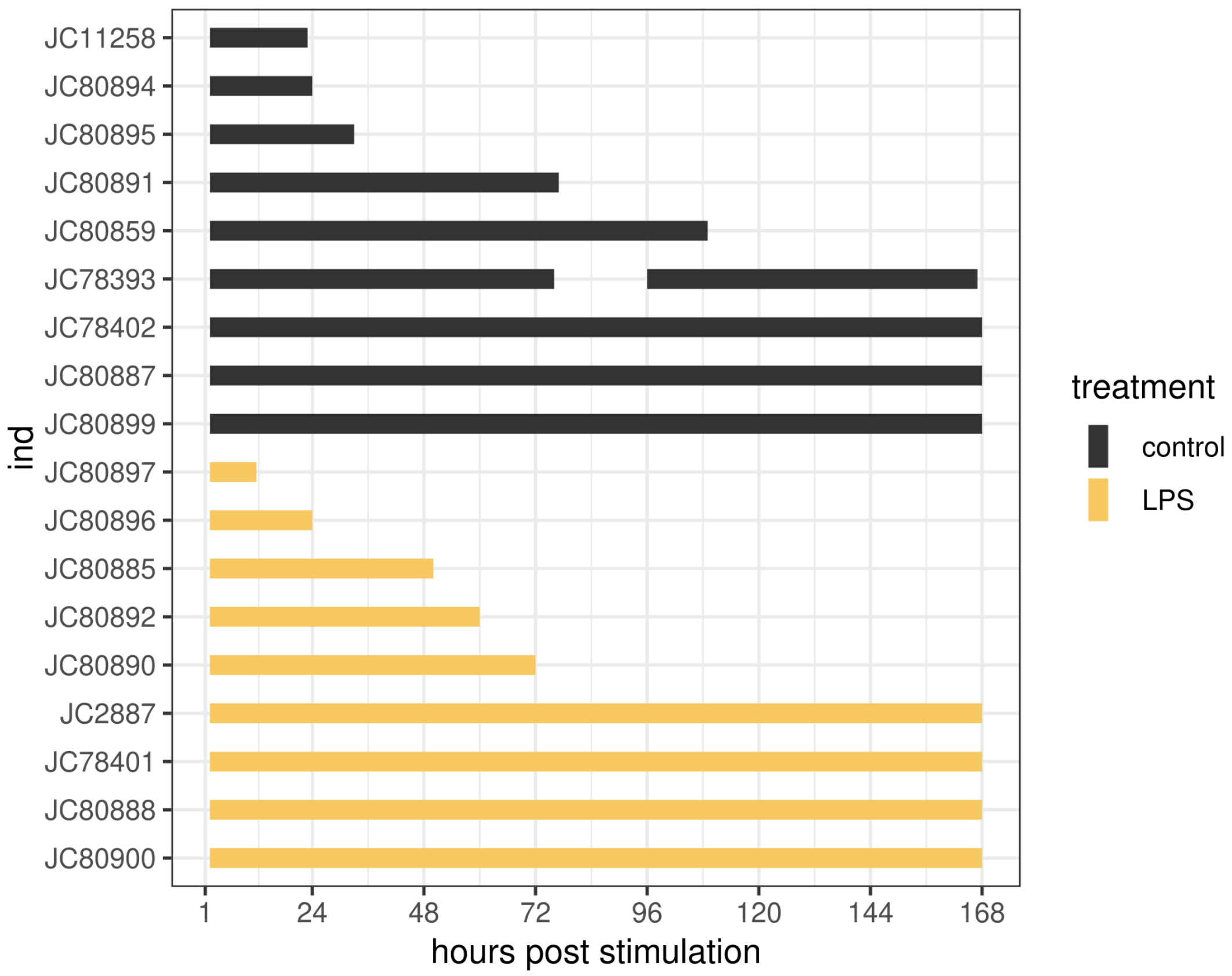


**Figure S9. Duration of available ACC data for the activity level analysis.** The availability of data decreased over the duration of the study due to rapidly decreasing battery levels in the loggers (Figure S3-4). In total, 25160 ACC bursts were recorded from the loggers. Individual JC78393 was recaptured during the study period whereas its initial biologger was exchanged for new fully changed one.

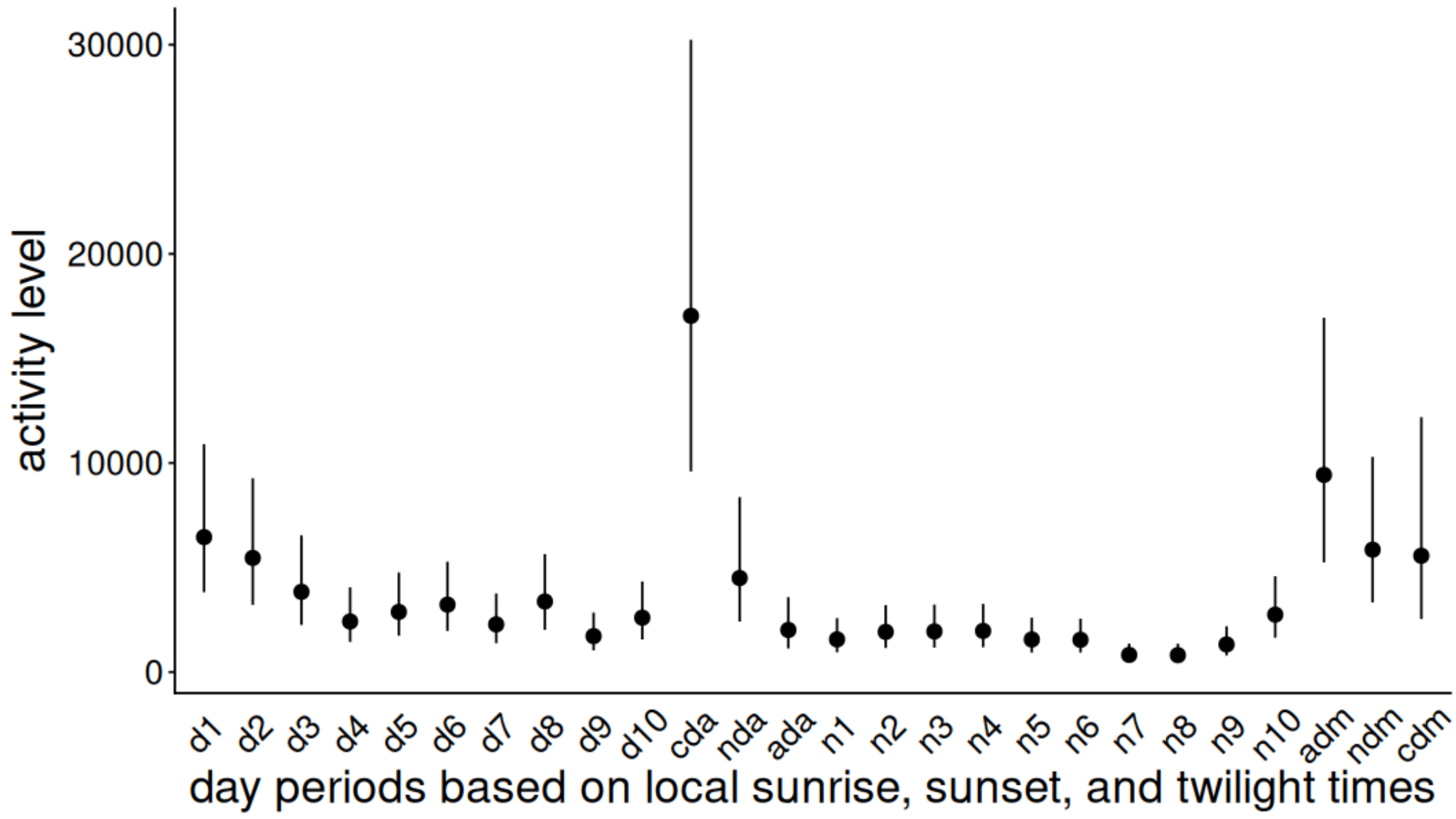


**Figure S10. Estimated activity level across different day periods.** Model-predicted mean activity level and 95% confidence interval for each day period. An activity value of 0 means no activity. The number of individuals with available logger data decreased from 18 to eight over the seven-day study period (Figure 1 and S9, and Table S1). In total, 1726 data points containing information on activity level per hour (calculated from 25160 ACC bursts) were included in the analysis. adm - Astronomical twilight morning, cda - Civil twilight evening, cdm - Civil twilight morning, nda - Nautical twilight evening, ndm - Nautical twilight morning, n - night, d - day

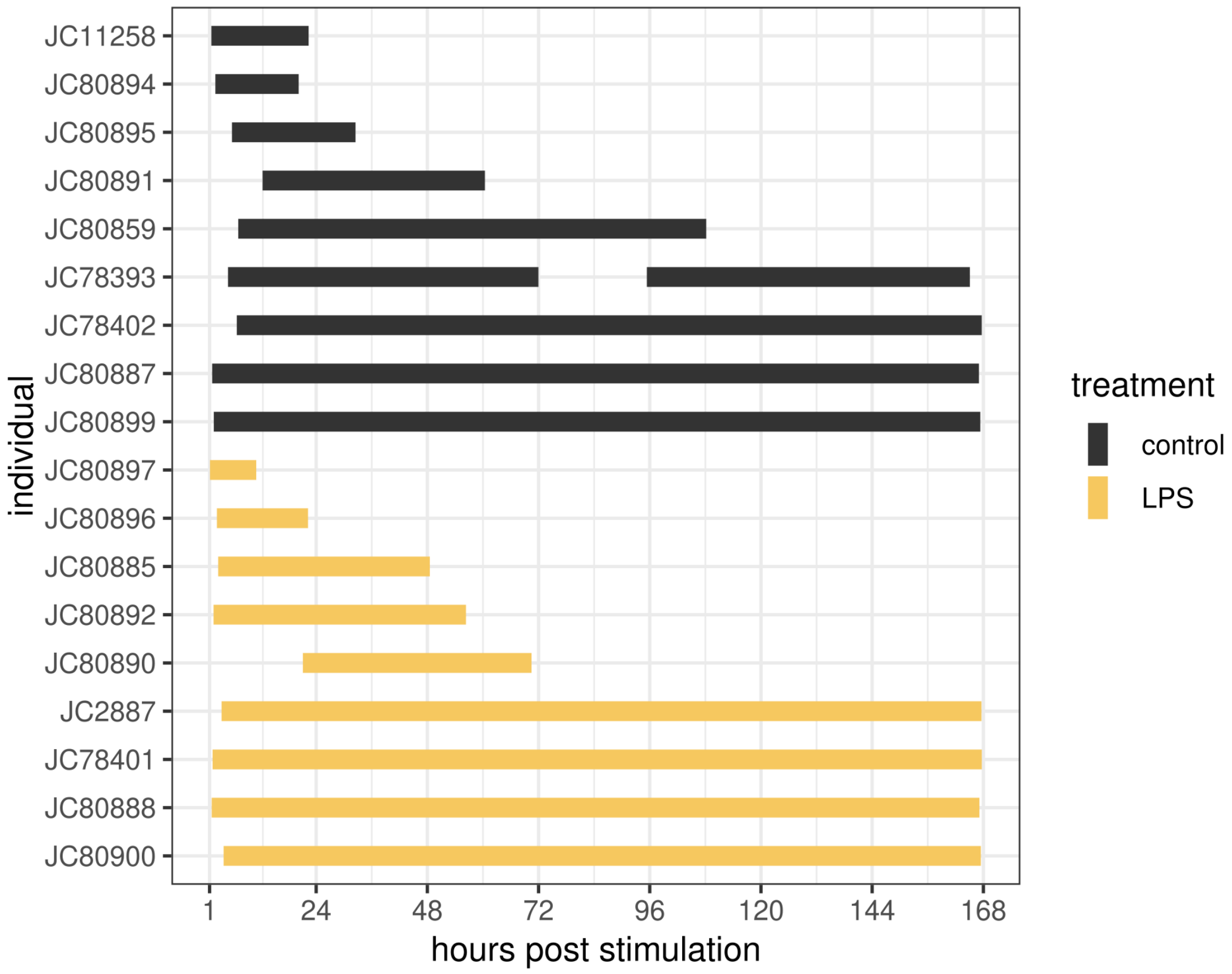


**Figure S11. Duration of available GPS data for the speed and habitat analysis.** Only GPS data with a Horizontal Dilution of Precision (HDOP) ≤ 2 were included in the habitat use analysis. The availability of data decreased over the duration of the study due to rapidly decreasing battery levels in the loggers (Figure S3-4). Individual JC78393 was recaptured during the study period whereas its initial biologger was exchanged for new fully changed one.

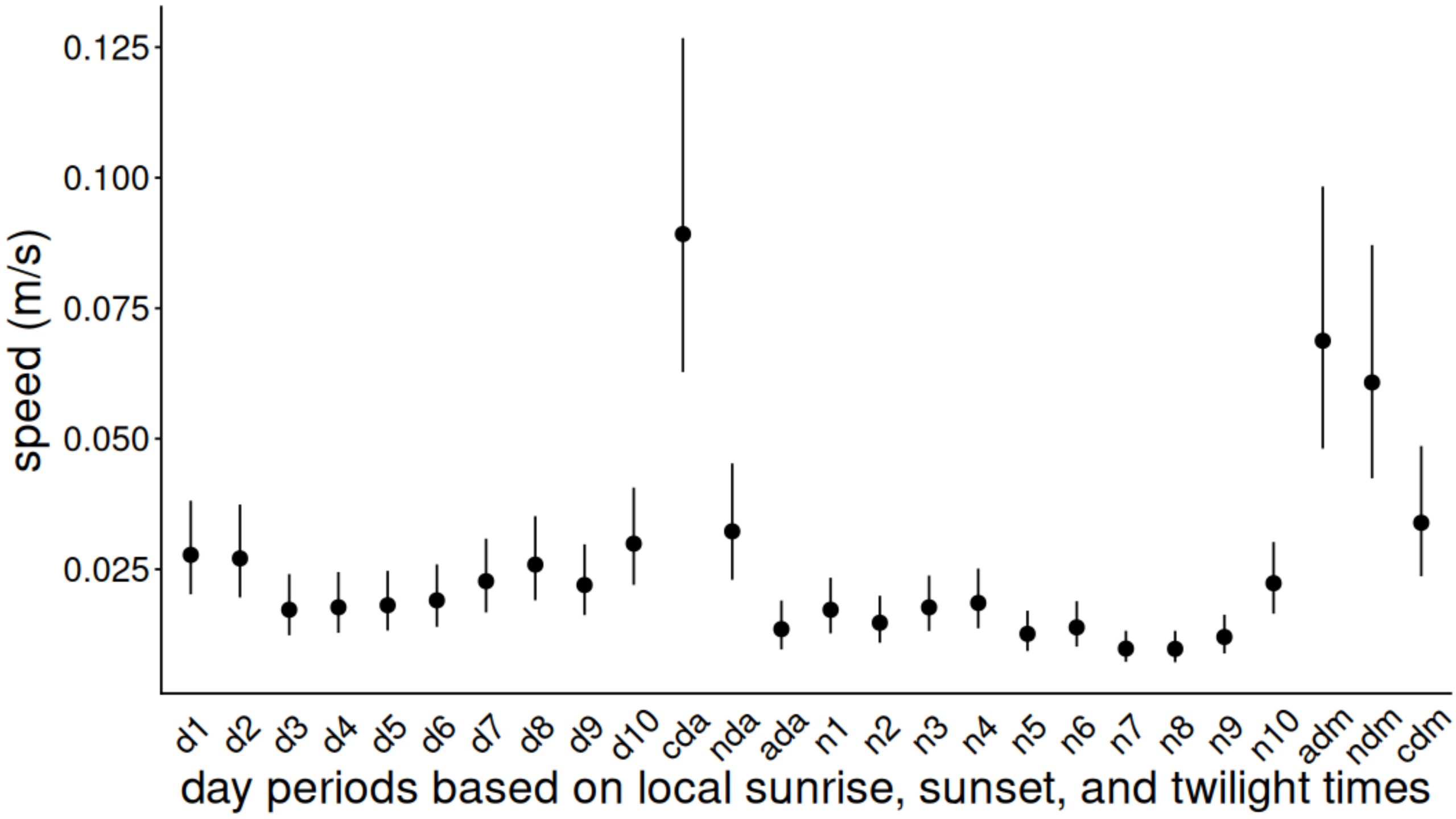


**Figure S12. Estimated speed across different day periods.** Model-predicted mean speed and 95% confidence interval for each day period. The number of individuals with available logger data decreased from 18 to eight over the seven-day study period (Figure 1 and S11, and Table S1). In total, 4378 high quality GPS points were included in the speed analysis. adm - Astronomical twilight morning, cda - Civil twilight evening, cdm - Civil twilight morning, nda - Nautical twilight evening, ndm - Nautical twilight morning, n - night, d - day

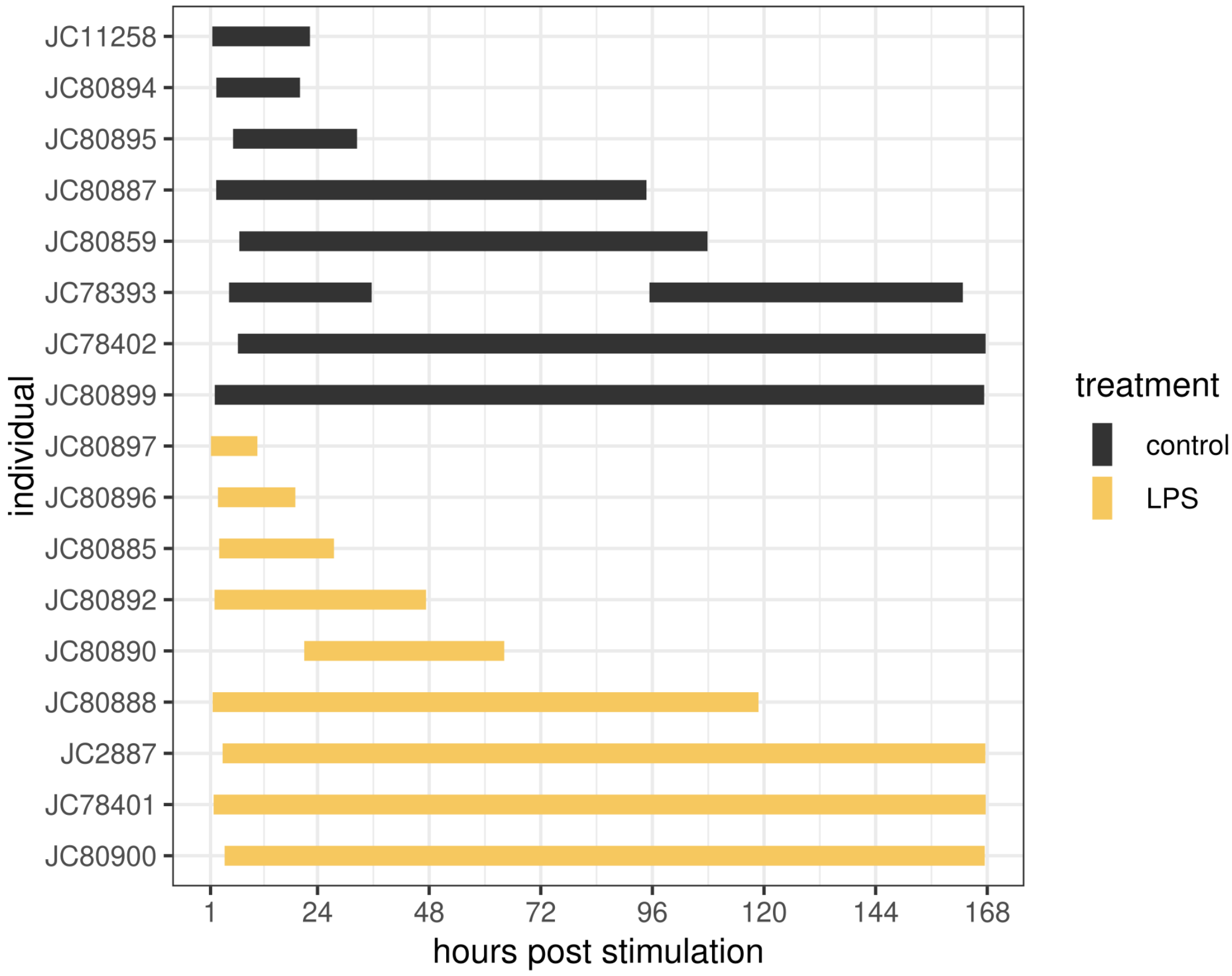


**Figure S13. Duration of available GPS data for the distance (step length) analysis.** Only GPS data with a Horizontal Dilution of Precision (HDOP) ≤ 2 and with distances measured between GPS fixes recorded 18 to 22 minutes apart were included in the distance analysis. In total, 3914 high quality GPS points remained for the distance analysis after filtering. The availability of data decreased over the duration of the study due to rapidly decreasing battery levels in the loggers (Figures S3-4). Individual JC78393 was recaptured during the study period whereas its initial biologger was exchanged for new fully changed one.

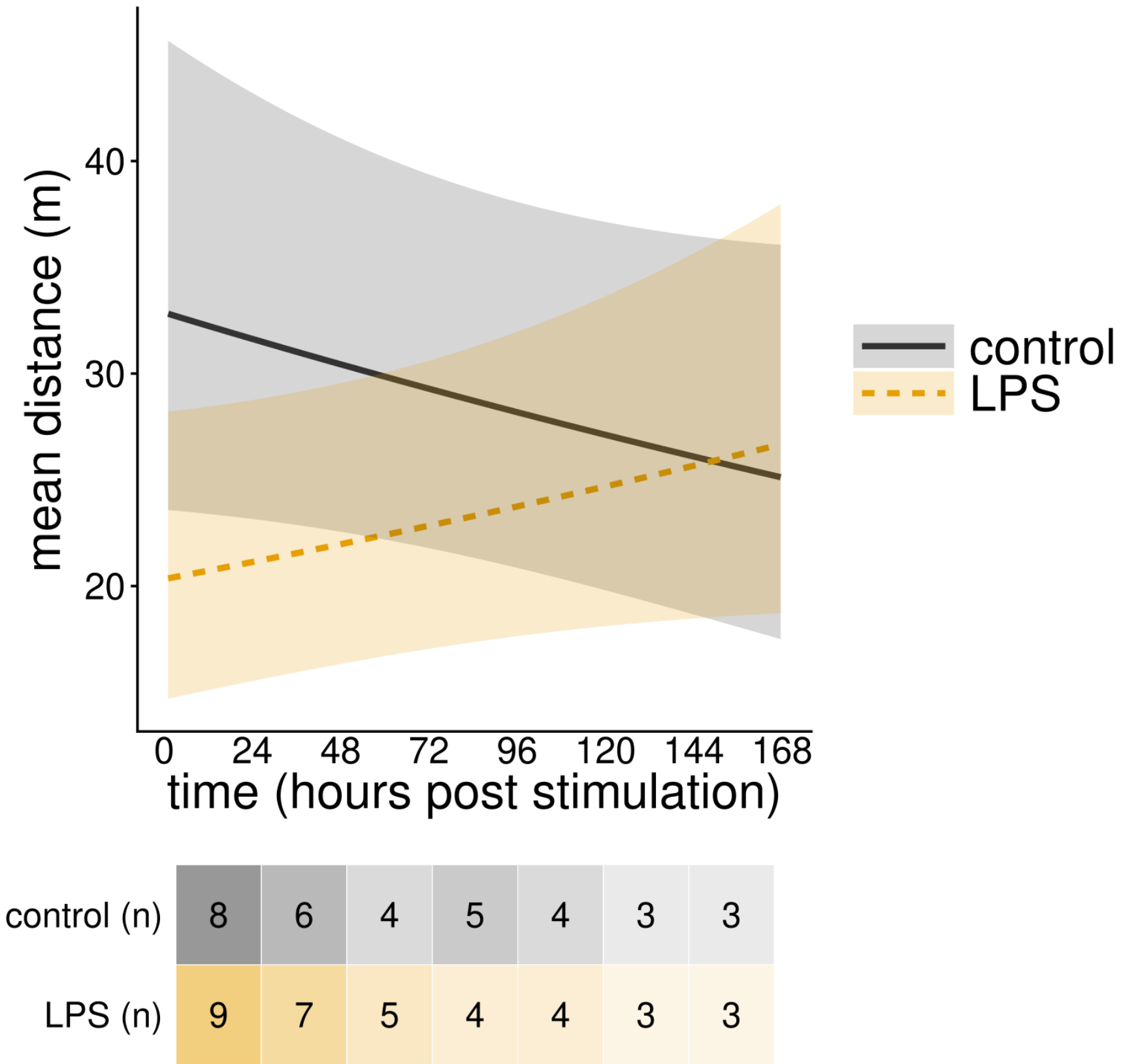


**Figure S14. Distance travelled (step length) following challenge with LPS stimulants.** Changes in distance travelled (meters per 20 minutes) measured remotely using bio-loggers. Model-predicted mean and 95% confidence interval for each treatment group is plotted until one week post stimulation. After filtering (HDOP > 2 and exclusion of intervals <18 min or >22 min), 3914 high-quality GPS locations remained for the distance analysis. The number of individuals contributing data declined from 8 to 3 in the control group and from 9 to 3 in the LPS treatment group (Figure S13). Numbers below the plot indicate the number of tracked individuals with at least one data point per day.

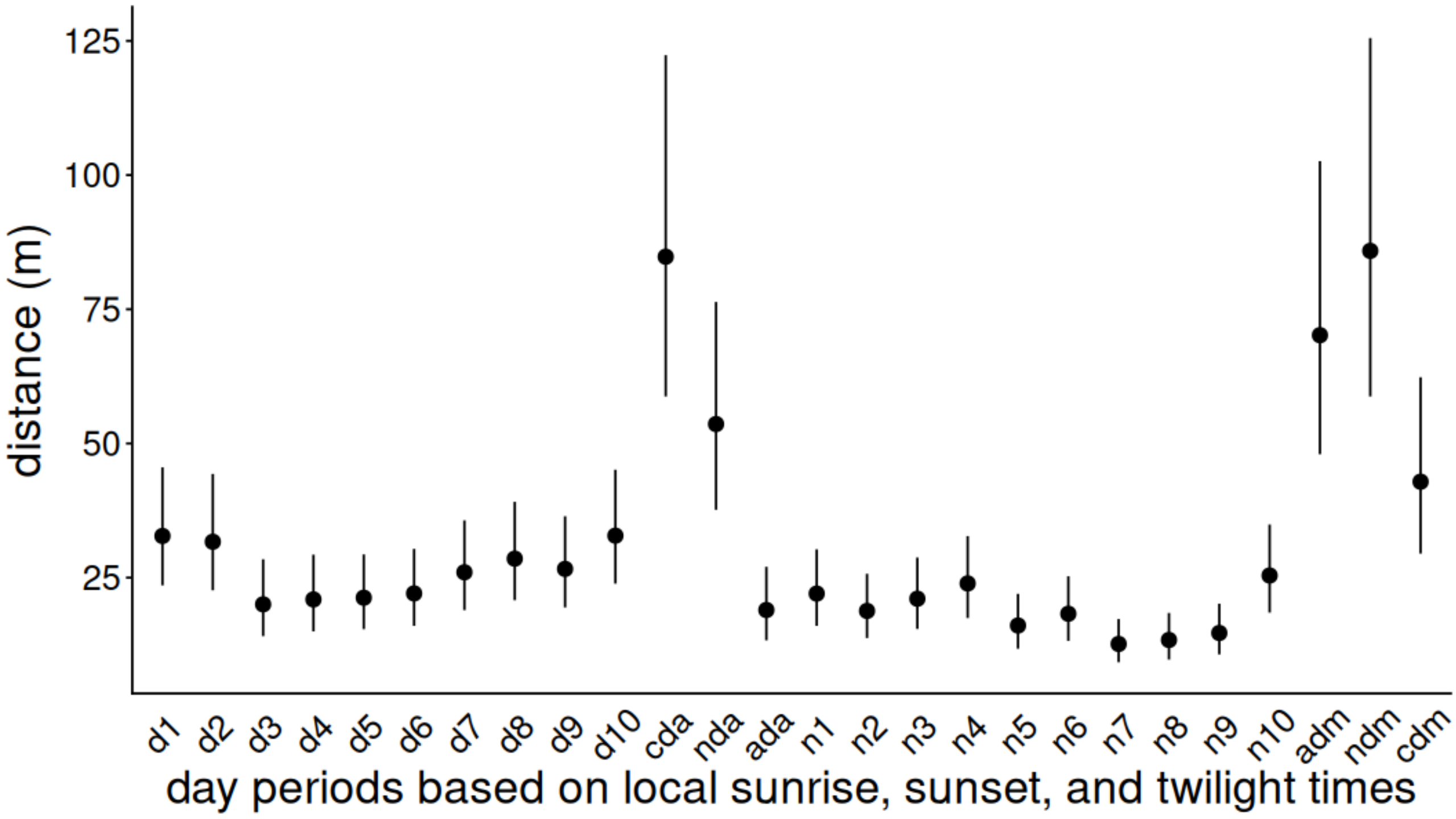


**Figure S15. Estimated distances travelled (meters per 20 minutes) across different day periods.** Model-predicted mean distance travelled (meters per 20 minutes) and 95% confidence interval for each day period. The number of individuals with available logger data decreased from 18 to six over the seven-day study period (Figure 1 and S13, and Table S1). After filtering (HDOP > 2 and exclusion of intervals <18 min or >22 min), 3914 high-quality GPS locations remained for the distance analysis. adm - Astronomical twilight morning, cda - Civil twilight evening, cdm - Civil twilight morning, nda - Nautical twilight evening, ndm - Nautical twilight morning, n - night, d - day

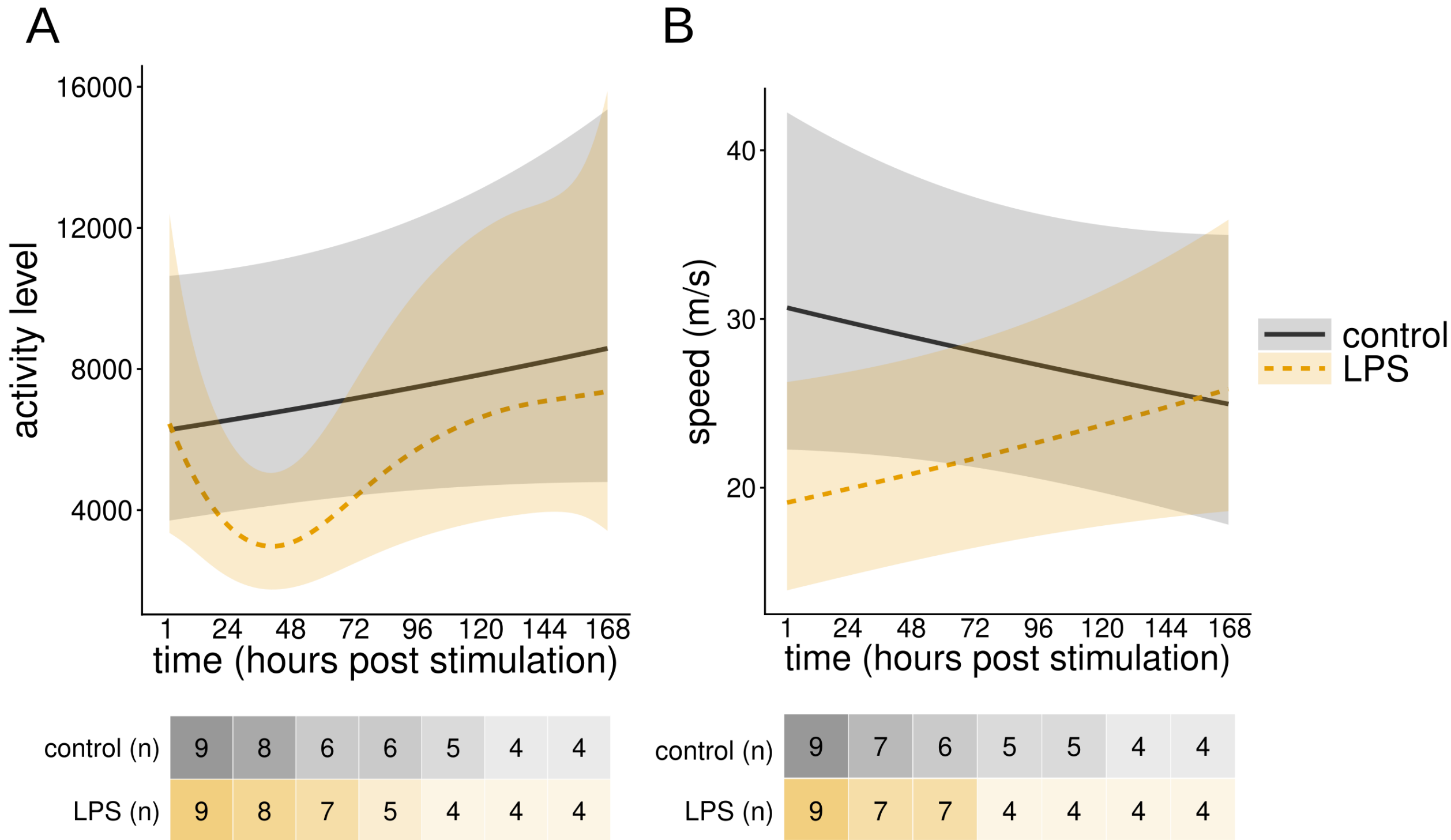


**Figure S16. Movement patterns following challenge with LPS stimulants: model predictions accounting for sex.** Changes in (A) activity level and (B) minimum speed (meters per second) were measured remotely using bio-loggers. This figure is based on generalized additive mixed models (GAMMs) fitted to the same datasets used for the original analyses, with sex included as an additional fixed effect. For the activity analysis, 1726 hourly data points containing information on activity level (calculated from 25,160 ACC bursts) were included in the model. For the speed analysis, 4378 high-quality GPS points remained after filtering. An activity value of 0 indicates no activity. Although the GAMMs were fitted using data from all individuals with sex included as an additional fixed effect, model predictions (mean ± 95% confidence intervals) are shown for males only (n = 16), as the number of female individuals contributing data was limited and did not allow for equally robust sex-specific visualization. Accordingly, the numbers below the plot indicate the number of male individuals contributing data on each day.

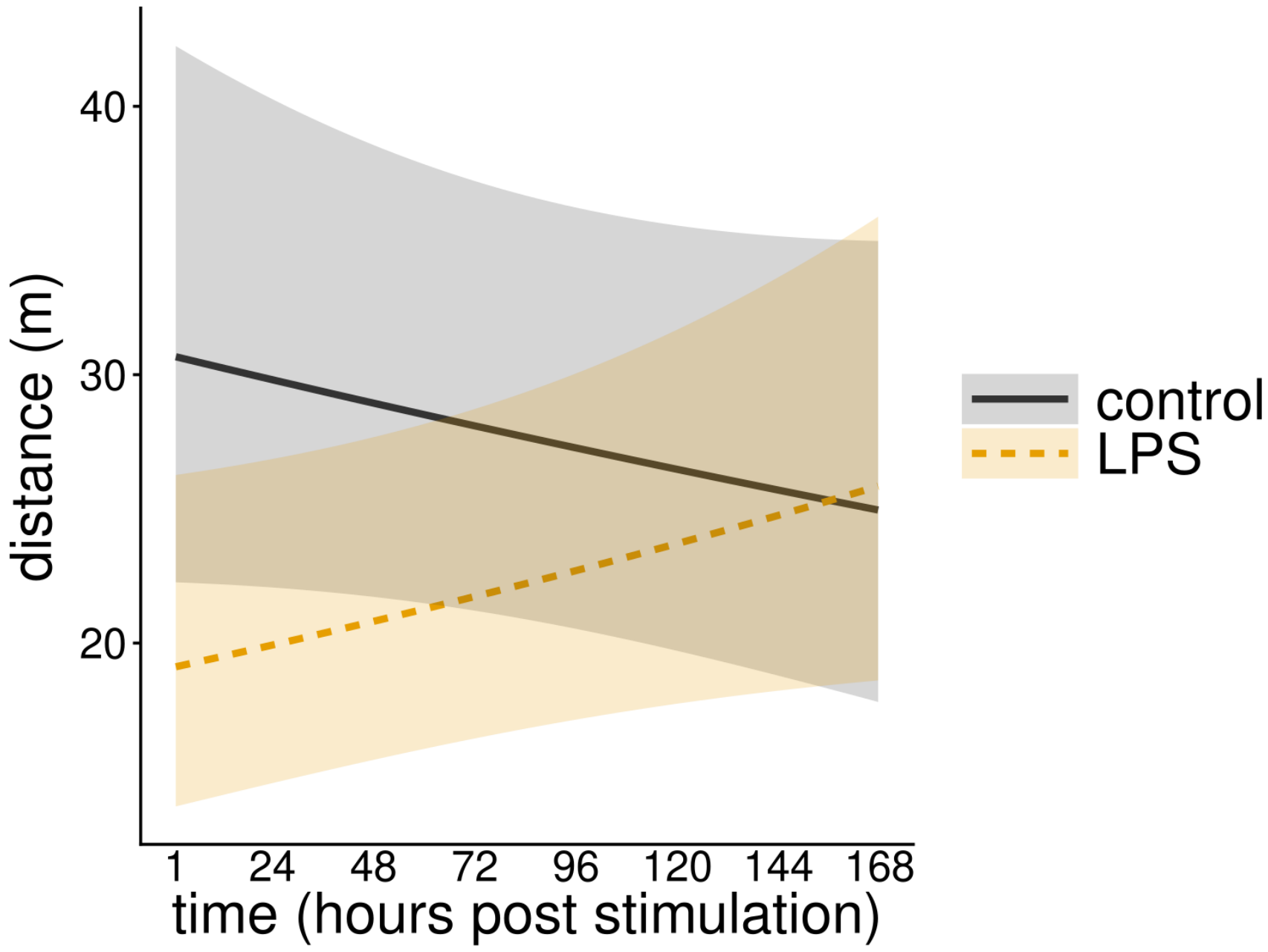


| | | | | | | | |
|---|---|---|---|---|---|---|---|
| control (n) | 8 | 6 | 4 | 5 | 4 | 3 | 3 |
| LPS (n) | 9 | 7 | 5 | 4 | 4 | 3 | 3 |

**Figure S17. Distance travelled (step length) following challenge with LPS stimulants: model predictions accounting for sex.** Changes in distance travelled (meters per 20 minutes) were measured remotely using bio-loggers. This figure is based on generalized additive mixed models (GAMMs) fitted to the same filtered GPS dataset used for the analysis shown in Figure S14, with sex included as an additional fixed effect. After filtering (HDOP > 2 and exclusion of intervals <18 min or >22 min), 3914 high-quality GPS locations from 17 individuals remained for the distance analysis. Predictions are plotted until one week post stimulation. Although the GAMM was fitted using data from all individuals with sex included as an additional fixed effect, model predictions (mean ± 95% confidence intervals) are shown for males only (n = 17), as the number of female individuals contributing data was limited and did not allow for equally robust sex-specific visualization. Accordingly, the numbers below the plot indicate the number of male individuals contributing data on each day.

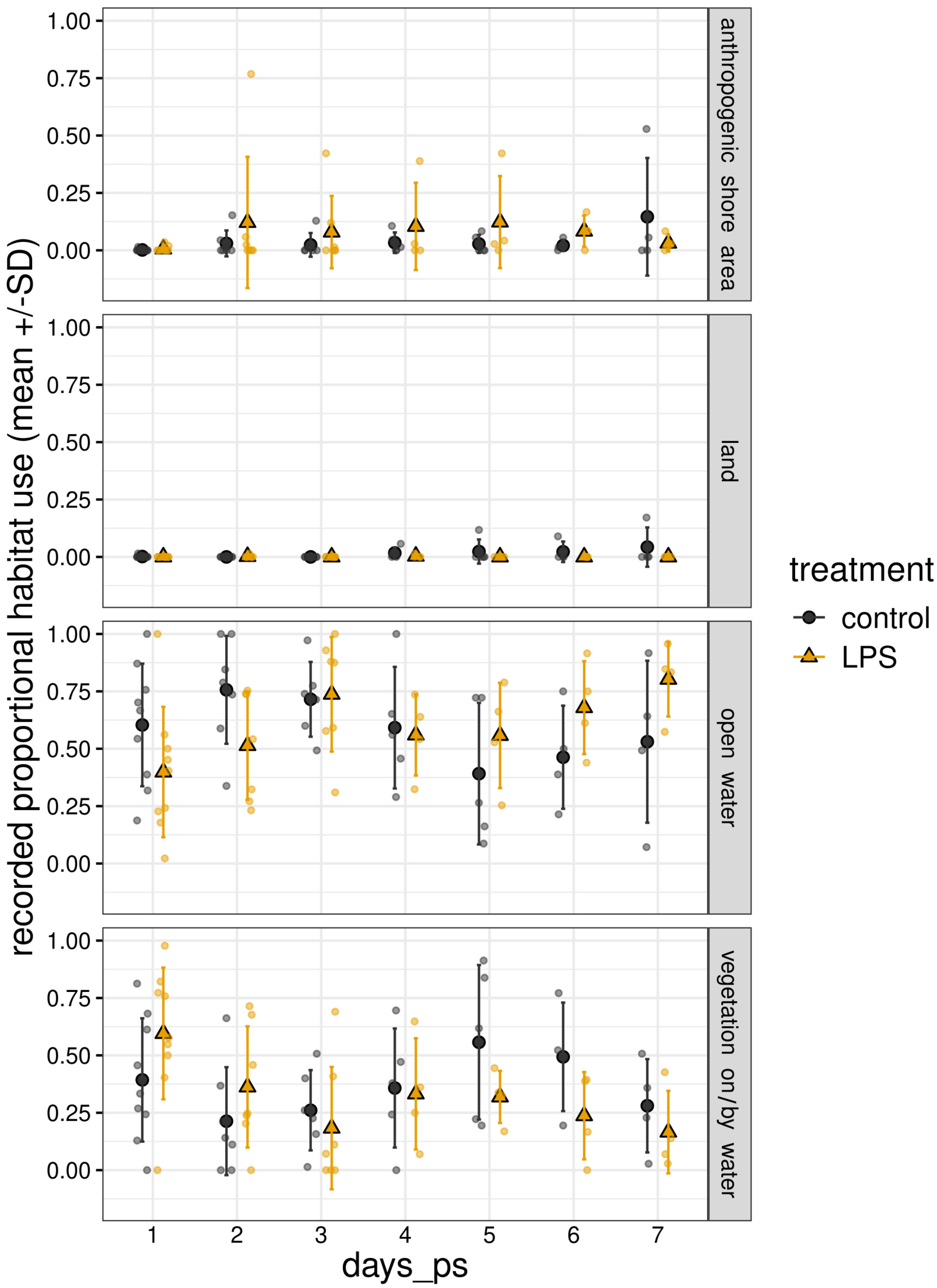


**Figure S18. Recorded proportional use of habitat types for each treatment group and time point** when using the land cover map from R.T.K.. Mean and standard deviation for the observed proportional use of each habitat for each treatment group and time point (days after stimulation). The observed proportional habitat use for each individual is shown as small circles.

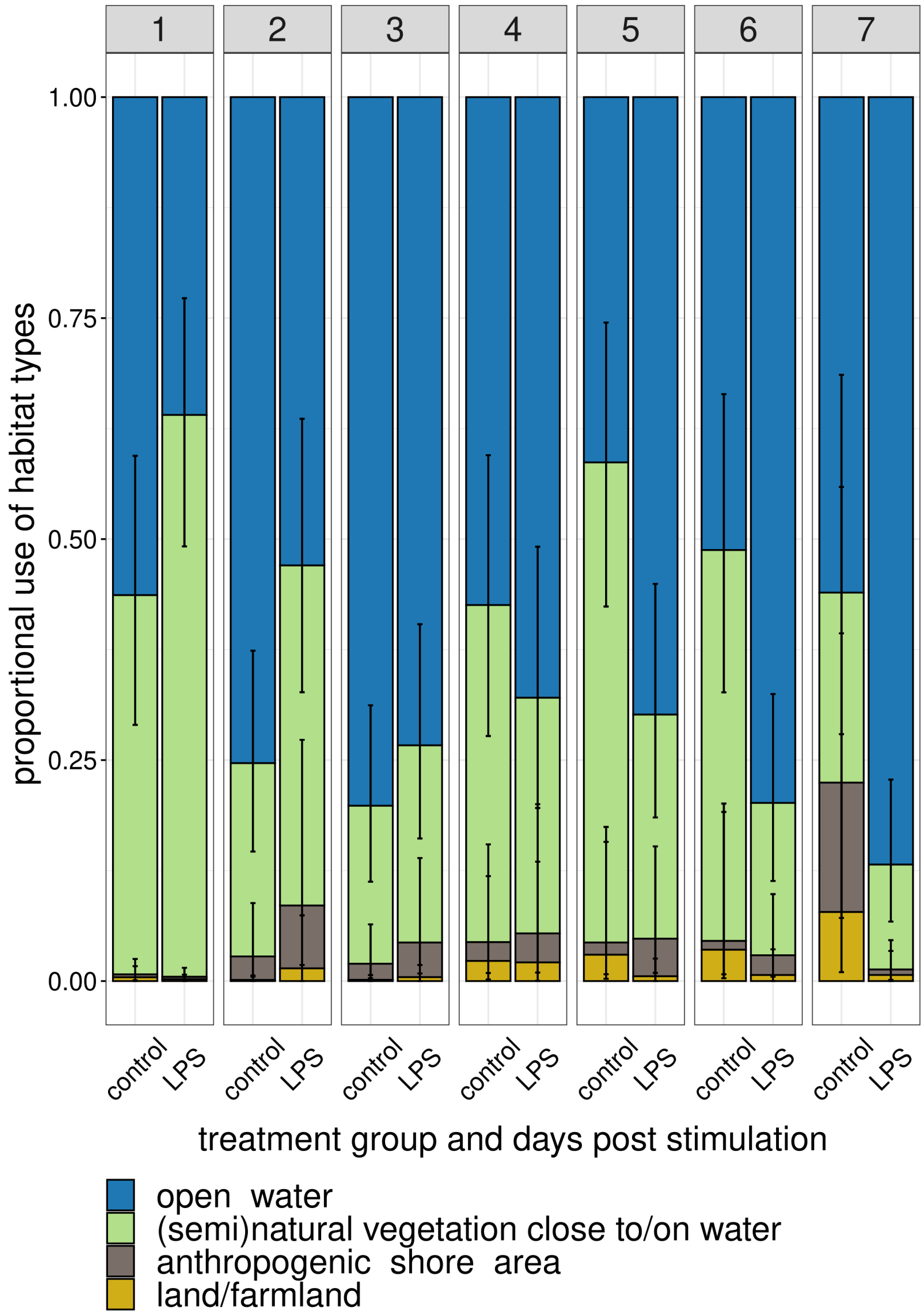


**Figure S19. Habitat use following challenge with LPS stimulants when using classification from E.J.** Cumulative mean proportion of the habitat types used by the mallards and cumulative 95% Credible Interval as estimated from the posterior distribution of the multinomial model when ran using the land cover map from E.J.. Habitat categories are shown in a consistent order from top to bottom as open water, (semi-)natural vegetation close to/on water, anthropogenic shore area, and land/farmland. The number of individuals included in the habitat use analysis decreased from 9 to 4 in each treatment group during the 7 days which the study took place (Figure S11). In total, habitat type information for 4394 GPS fixes (HDOP≤2) was included in the analysis.

## Supplementary tables

**Table S1.** Overview of study inclusion period and logger data availability for each individual included in the main study.
start and end = time and date when the first and last data point was transmitted from each bio-logger.
hours of data = hours between the first and last transmitted data point from each bio-logger. datapoints GPS = the total number of transmitted high-quality GPS fixes per bio-logger
datapoints ACC_agg = the number of hours with aggregated acceleration (ACC) data available for each individual
* Individual JC78393 was recaptured during the study period and equipped with a fully charged replacement logger. Therefore, the number of GPS fixes and aggregated ACC records are presented separately for each logger deployment.

| individual | device id | treatment | sex | start | end | hours of data | datapoints GPS | datapointsACC_agg |
|---|---|---|---|---|---|---|---|---|
| JC80882 | 182892 | LPS | F | 2019-01-28 10:00:00 | | 0 | 0 | 0 |
| JC80888 | 171010 | LPS | F | 2019-01-29 10:57:14 | 2019-02-05 09:19:35 | 166.4 | 423 | 167 |
| JC80887 | 180090 | control | M | 2019-01-29 11:23:05 | 2019-02-05 09:33:35 | 166.2 | 383 | 167 |
| JC80889 | 182922 | control | F | 2019-01-30 10:30:00 | | 0 | 0 | 0 |
| JC80885 | 182934 | LPS | M | 2019-01-30 11:21:16 | 2019-02-01 09:41:56 | 46.3 | 101 | 49 |
| JC80891 | 182898 | control | M | 2019-01-30 21:13:59 | 2019-02-02 05:13:59 | 56.0 | 10 | 71 |
| JC80890 | 182926 | LPS | M | 2019-01-31 06:17:53 | 2019-02-02 08:17:50 | 50.0 | 126 | 71 |
| JC80892 | 182899 | LPS | M | 2019-01-31 11:26:08 | 2019-02-02 18:35:35 | 55.2 | 148 | 59 |
| JC11258 | 182924 | control | M | 2019-02-05 10:27:15 | 2019-02-06 07:46:20 | 21.3 | 62 | 22 |
| JC80894 | 182920 | control | M | 2019-02-05 11:11:20 | 2019-02-06 05:30:07 | 18.3 | 37 | 23 |
| JC80896 | 182919 | LPS | M | 2019-02-06 11:27:44 | 2019-02-07 07:47:43 | 20.3 | 44 | 23 |
| JC80895 | 182918 | control | M | 2019-02-06 15:04:43 | 2019-02-07 18:04:03 | 27.0 | 71 | 32 |
| JC80897 | 182917 | LPS | M | 2019-02-07 11:13:59 | 2019-02-07 21:32:50 | 10.3 | 28 | 11 |
| JC78393* | 182925 | control | M | 2019-02-07 15:15:11 | 2019-02-10 14:15:04 | 71.0 | 130 | 75 |
| JC80859 | 182896 | control | F | 2019-02-07 17:14:39 | 2019-02-11 22:55:05 | 101.7 | 288 | 108 |
| JC78393* | 182895 | control | M | 2019-02-11 10:38:02 | 2019-02-14 08:02:33 | 69.4 | 169 | 72 |
| JC80899 | 182901 | control | M | 2019-02-14 10:49:02 | 2019-02-21 08:35:43 | 165.8 | 484 | 167 |
| JC2887 | 182893 | LPS | M | 2019-02-14 12:35:05 | 2019-02-21 08:55:59 | 164.3 | 473 | 167 |
| JC80900 | 182900 | LPS | M | 2019-02-15 12:16:05 | 2019-02-22 08:02:41 | 163.8 | 459 | 167 |
| JC78401 | 182904 | LPS | M | 2019-02-20 | 2019-02-27 | 166.3 | 490 | 167 |

| | | | | | | | | |
|---|---|---|---|---|---|---|---|---|
| | | | | 10:31:08 | 08:51:24 | | | |
| JC78402 | 182897 | control | M | 2019-02-20 15:37:09 | 2019-02-27 08:43:02 | 161.1 | 468 | 167 |

**Table S2.** Habitats included in the four habitat type categories.

| Habitat class | Habitats included in class |
|---|---|
| open water | open water, artificial water body, streams |
| (semi)natural vegetation close to/on water | forest close to water, reed bed, forested marsh, marsh, logs on water, trees overhanging water, pond lilies |
| anthropogenic shore area | urban area, populated lakeside |
| land/farmland | farmland, grassland |

**Table S3.** Mean proportion of the habitat types used by the mallards and 95% Credible Interval for each day post stimulation and treatment group as estimated from the posterior distribution of the multinomial model. Results from the multinomial model when using the land cover map from R.T.K. (results shown in manuscript).

| | treat_days | habitat_type | mean | sd | 2.5% | 97.5% | Rhat | n.eff |
|---|---|---|---|---|---|---|---|---|
| pfit[1,1] | control 1 | open_water | 0.570 | 0.078 | 0.410 | 0.716 | 1.001 | 63000 |
| pfit[1,2] | control 1 | vegetation_on_by_water | 0.424 | 0.078 | 0.276 | 0.584 | 1.001 | 23000 |
| pfit[1,3] | control 1 | anthropogenic_shore_area | 0.002 | 0.003 | 0.000 | 0.008 | 1.001 | 54000 |
| pfit[1,4] | control 1 | land | 0.004 | 0.026 | 0.000 | 0.015 | 1.001 | 7900 |
| pfit[2,1] | control 2 | open_water | 0.765 | 0.057 | 0.639 | 0.863 | 1.001 | 40000 |
| pfit[2,2] | control 2 | vegetation_on_by_water | 0.204 | 0.055 | 0.112 | 0.326 | 1.001 | 18000 |
| pfit[2,3] | control 2 | anthropogenic_shore_area | 0.029 | 0.027 | 0.005 | 0.099 | 1.001 | 34000 |
| pfit[2,4] | control 2 | land | 0.001 | 0.011 | 0.000 | 0.006 | 1.001 | 76000 |
| pfit[3,1] | control 3 | open_water | 0.812 | 0.050 | 0.699 | 0.894 | 1.001 | 86000 |
| pfit[3,2] | control 3 | vegetation_on_by_water | 0.167 | 0.048 | 0.088 | 0.276 | 1.001 | 20000 |
| pfit[3,3] | control 3 | anthropogenic_shore_area | 0.020 | 0.020 | 0.003 | 0.071 | 1.001 | 58000 |
| pfit[3,4] | control 3 | land | 0.001 | 0.011 | 0.000 | 0.006 | 1.001 | 140000 |
| pfit[4,1] | control 4 | open_water | 0.576 | 0.084 | 0.405 | 0.723 | 1.007 | 8800 |
| pfit[4,2] | control 4 | vegetation_on_by_water | 0.375 | 0.083 | 0.220 | 0.542 | 1.003 | 5700 |
| pfit[4,3] | control 4 | anthropogenic_shore_area | 0.023 | 0.023 | 0.003 | 0.082 | 1.001 | 39000 |
| pfit[4,4] | control 4 | land | 0.025 | 0.064 | 0.002 | 0.122 | 1.001 | 5600 |
| pfit[5,1] | control 5 | open_water | 0.430 | 0.084 | 0.267 | 0.592 | 1.004 | 15000 |
| pfit[5,2] | control 5 | vegetation_on_by_water | 0.519 | 0.092 | 0.332 | 0.686 | 1.006 | 3900 |
| pfit[5,3] | control 5 | anthropogenic_shore_area | 0.015 | 0.017 | 0.002 | 0.057 | 1.001 | 23000 |
| pfit[5,4] | control 5 | land | 0.035 | 0.075 | 0.004 | 0.176 | 1.001 | 6800 |
| pfit[6,1] | control 6 | open_water | 0.540 | 0.087 | 0.365 | 0.696 | 1.006 | 10000 |
| pfit[6,2] | control 6 | vegetation_on_by_water | 0.417 | 0.087 | 0.251 | 0.588 | 1.004 | 5300 |
| pfit[6,3] | control 6 | anthropogenic_shore_area | 0.014 | 0.016 | 0.002 | 0.055 | 1.001 | 34000 |
| pfit[6,4] | control 6 | land | 0.029 | 0.068 | 0.003 | 0.142 | 1.001 | 8400 |
| pfit[7,1] | control 7 | open_water | 0.586 | 0.105 | 0.335 | 0.746 | 1.009 | 4300 |
| pfit[7,2] | control 7 | vegetation_on_by_water | 0.195 | 0.063 | 0.084 | 0.332 | 1.003 | 4600 |
| pfit[7,3] | control 7 | anthropogenic_shore_area | 0.160 | 0.105 | 0.032 | 0.433 | 1.001 | 19000 |
| pfit[7,4] | control 7 | land | 0.059 | 0.094 | 0.007 | 0.292 | 1.001 | 6800 |
| pfit[8,1] | LPS 1 | open_water | 0.352 | 0.072 | 0.219 | 0.500 | 1.001 | 55000 |
| pfit[8,2] | LPS 1 | vegetation_on_by_water | 0.644 | 0.073 | 0.492 | 0.778 | 1.001 | 22000 |
| pfit[8,3] | LPS 1 | anthropogenic_shore_area | 0.003 | 0.004 | 0.000 | 0.012 | 1.001 | 22000 |
| pfit[8,4] | LPS 1 | land | 0.002 | 0.021 | 0.000 | 0.007 | 1.001 | 21000 |
| pfit[9,1] | LPS 2 | open_water | 0.540 | 0.083 | 0.373 | 0.683 | 1.002 | 12000 |
| pfit[9,2] | LPS 2 | vegetation_on_by_water | 0.373 | 0.081 | 0.220 | 0.536 | 1.001 | 11000 |
| pfit[9,3] | LPS 2 | anthropogenic_shore_area | 0.075 | 0.057 | 0.015 | 0.224 | 1.001 | 35000 |
| pfit[9,4] | LPS 2 | land | 0.012 | 0.068 | 0.000 | 0.054 | 1.001 | 42000 |
| pfit[10,1] | LPS 3 | open_water | 0.733 | 0.066 | 0.592 | 0.839 | 1.003 | 17000 |
| pfit[10,2] | LPS 3 | vegetation_on_by_water | 0.221 | 0.058 | 0.122 | 0.350 | 1.001 | 28000 |
| pfit[10,3] | LPS 3 | anthropogenic_shore_area | 0.043 | 0.036 | 0.008 | 0.135 | 1.001 | 15000 |
| pfit[10,4] | LPS 3 | land | 0.004 | 0.033 | 0.000 | 0.015 | 1.001 | 140000 |
| pfit[11,1] | LPS 4 | open_water | 0.682 | 0.084 | 0.515 | 0.806 | 1.004 | 13000 |
| pfit[11,2] | LPS 4 | vegetation_on_by_water | 0.266 | 0.069 | 0.144 | 0.412 | 1.002 | 13000 |
| pfit[11,3] | LPS 4 | anthropogenic_shore_area | 0.034 | 0.031 | 0.006 | 0.111 | 1.001 | 29000 |
| pfit[11,4] | LPS 4 | land | 0.018 | 0.076 | 0.000 | 0.104 | 1.001 | 34000 |
| pfit[12,1] | LPS 5 | open_water | 0.691 | 0.072 | 0.540 | 0.811 | 1.002 | 23000 |
| pfit[12,2] | LPS 5 | vegetation_on_by_water | 0.260 | 0.066 | 0.145 | 0.403 | 1.001 | 20000 |

| | | | | | | | | |
|---|---|---|---|---|---|---|---|---|
| pfit[12,3] | LPS 5 | anthropogenic_shore_area | 0.044 | 0.038 | 0.008 | 0.141 | 1.001 | 19000 |
| pfit[12,4] | LPS 5 | land | 0.005 | 0.036 | 0.000 | 0.022 | 1.001 | 140000 |
| pfit[13,1] | LPS 6 | open_water | 0.790 | 0.062 | 0.663 | 0.882 | 1.004 | 19000 |
| pfit[13,2] | LPS 6 | vegetation_on_by_water | 0.181 | 0.052 | 0.095 | 0.298 | 1.001 | 35000 |
| pfit[13,3] | LPS 6 | anthropogenic_shore_area | 0.023 | 0.022 | 0.004 | 0.077 | 1.001 | 13000 |
| pfit[13,4] | LPS 6 | land | 0.006 | 0.040 | 0.000 | 0.030 | 1.001 | 77000 |
| pfit[14,1] | LPS 7 | open_water | 0.870 | 0.051 | 0.774 | 0.933 | 1.010 | 22000 |
| pfit[14,2] | LPS 7 | vegetation_on_by_water | 0.117 | 0.038 | 0.058 | 0.205 | 1.001 | 86000 |
| pfit[14,3] | LPS 7 | anthropogenic_shore_area | 0.007 | 0.008 | 0.001 | 0.026 | 1.001 | 20000 |
| pfit[14,4] | LPS 7 | land | 0.006 | 0.039 | 0.000 | 0.028 | 1.001 | 29000 |

**Table S4.** Mean proportion of the habitat types used by the mallards and 95% Credible Interval for each day post stimulation and treatment group as estimated from the posterior distribution of the multinomial model. Results from the multinomial model when using the land cover map from E.J. (results shown for comparison to those in main manuscript).

| | treat_days | habitat_type | mean | sd | 2.5% | 97.5% | Rhat | n.eff |
|---|---|---|---|---|---|---|---|---|
| pfit[1,1] | control 1 | open_water | 0.563 | 0.078 | 0.406 | 0.710 | 1.001 | 140000 |
| pfit[1,2] | control 1 | vegetation_on_by_water | 0.429 | 0.078 | 0.281 | 0.586 | 1.001 | 33000 |
| pfit[1,3] | control 1 | anthropogenic_shore_area | 0.003 | 0.004 | 0.000 | 0.013 | 1.001 | 43000 |
| pfit[1,4] | control 1 | land | 0.004 | 0.026 | 0.000 | 0.017 | 1.001 | 16000 |
| pfit[2,1] | control 2 | open_water | 0.753 | 0.058 | 0.626 | 0.853 | 1.001 | 110000 |
| pfit[2,2] | control 2 | vegetation_on_by_water | 0.219 | 0.057 | 0.121 | 0.344 | 1.001 | 140000 |
| pfit[2,3] | control 2 | anthropogenic_shore_area | 0.027 | 0.023 | 0.005 | 0.085 | 1.001 | 33000 |
| pfit[2,4] | control 2 | land | 0.001 | 0.010 | 0.000 | 0.006 | 1.001 | 39000 |
| pfit[3,1] | control 3 | open_water | 0.802 | 0.051 | 0.688 | 0.888 | 1.001 | 60000 |
| pfit[3,2] | control 3 | vegetation_on_by_water | 0.179 | 0.050 | 0.095 | 0.290 | 1.001 | 65000 |
| pfit[3,3] | control 3 | anthropogenic_shore_area | 0.018 | 0.017 | 0.003 | 0.060 | 1.001 | 19000 |
| pfit[3,4] | control 3 | land | 0.001 | 0.011 | 0.000 | 0.007 | 1.001 | 110000 |
| pfit[4,1] | control 4 | open_water | 0.575 | 0.083 | 0.405 | 0.723 | 1.006 | 17000 |
| pfit[4,2] | control 4 | vegetation_on_by_water | 0.381 | 0.082 | 0.226 | 0.546 | 1.002 | 12000 |
| pfit[4,3] | control 4 | anthropogenic_shore_area | 0.021 | 0.019 | 0.003 | 0.071 | 1.001 | 21000 |
| pfit[4,4] | control 4 | land | 0.023 | 0.062 | 0.002 | 0.119 | 1.001 | 12000 |
| pfit[5,1] | control 5 | open_water | 0.413 | 0.083 | 0.255 | 0.576 | 1.003 | 26000 |
| pfit[5,2] | control 5 | vegetation_on_by_water | 0.543 | 0.091 | 0.356 | 0.706 | 1.006 | 8000 |
| pfit[5,3] | control 5 | anthropogenic_shore_area | 0.014 | 0.014 | 0.002 | 0.050 | 1.001 | 12000 |
| pfit[5,4] | control 5 | land | 0.030 | 0.070 | 0.003 | 0.158 | 1.001 | 14000 |
| pfit[6,1] | control 6 | open_water | 0.512 | 0.088 | 0.336 | 0.673 | 1.005 | 12000 |
| pfit[6,2] | control 6 | vegetation_on_by_water | 0.442 | 0.089 | 0.266 | 0.612 | 1.004 | 11000 |
| pfit[6,3] | control 6 | anthropogenic_shore_area | 0.010 | 0.011 | 0.001 | 0.039 | 1.001 | 26000 |
| pfit[6,4] | control 6 | land | 0.035 | 0.076 | 0.003 | 0.191 | 1.001 | 25000 |
| pfit[7,1] | control 7 | open_water | 0.561 | 0.103 | 0.314 | 0.721 | 1.008 | 8800 |
| pfit[7,2] | control 7 | vegetation_on_by_water | 0.215 | 0.068 | 0.093 | 0.359 | 1.003 | 9500 |
| pfit[7,3] | control 7 | anthropogenic_shore_area | 0.146 | 0.094 | 0.027 | 0.390 | 1.001 | 11000 |
| pfit[7,4] | control 7 | land | 0.078 | 0.110 | 0.010 | 0.393 | 1.001 | 11000 |
| pfit[8,1] | LPS 1 | open_water | 0.360 | 0.072 | 0.228 | 0.508 | 1.001 | 43000 |
| pfit[8,2] | LPS 1 | vegetation_on_by_water | 0.636 | 0.073 | 0.484 | 0.769 | 1.005 | 14000 |
| pfit[8,3] | LPS 1 | anthropogenic_shore_area | 0.003 | 0.003 | 0.000 | 0.011 | 1.002 | 3600 |
| pfit[8,4] | LPS 1 | land | 0.002 | 0.024 | 0.000 | 0.007 | 1.001 | 140000 |
| pfit[9,1] | LPS 2 | open_water | 0.530 | 0.084 | 0.364 | 0.673 | 1.013 | 11000 |
| pfit[9,2] | LPS 2 | vegetation_on_by_water | 0.385 | 0.083 | 0.227 | 0.546 | 1.008 | 9300 |
| pfit[9,3] | LPS 2 | anthropogenic_shore_area | 0.071 | 0.054 | 0.014 | 0.214 | 1.002 | 1800 |
| pfit[9,4] | LPS 2 | land | 0.014 | 0.077 | 0.000 | 0.074 | 1.001 | 51000 |
| pfit[10,1] | LPS 3 | open_water | 0.733 | 0.065 | 0.596 | 0.839 | 1.004 | 140000 |
| pfit[10,2] | LPS 3 | vegetation_on_by_water | 0.223 | 0.058 | 0.124 | 0.351 | 1.001 | 28000 |
| pfit[10,3] | LPS 3 | anthropogenic_shore_area | 0.039 | 0.033 | 0.007 | 0.124 | 1.002 | 2200 |
| pfit[10,4] | LPS 3 | land | 0.004 | 0.035 | 0.000 | 0.018 | 1.001 | 94000 |
| pfit[11,1] | LPS 4 | open_water | 0.679 | 0.088 | 0.509 | 0.804 | 1.013 | 8000 |
| pfit[11,2] | LPS 4 | vegetation_on_by_water | 0.267 | 0.069 | 0.143 | 0.411 | 1.005 | 9300 |
| pfit[11,3] | LPS 4 | anthropogenic_shore_area | 0.033 | 0.029 | 0.006 | 0.106 | 1.002 | 2000 |
| pfit[11,4] | LPS 4 | land | 0.021 | 0.086 | 0.000 | 0.135 | 1.001 | 140000 |
| pfit[12,1] | LPS 5 | open_water | 0.699 | 0.071 | 0.551 | 0.815 | 1.006 | 47000 |
| pfit[12,2] | LPS 5 | vegetation_on_by_water | 0.254 | 0.064 | 0.142 | 0.393 | 1.001 | 21000 |
| pfit[12,3] | LPS 5 | anthropogenic_shore_area | 0.043 | 0.036 | 0.008 | 0.134 | 1.002 | 2100 |

| | | | | | | | | |
|---|---|---|---|---|---|---|---|---|
| pfit[12,4] | LPS 5 | land | 0.005 | 0.039 | 0.000 | 0.025 | 1.001 | 140000 |
| pfit[13,1] | LPS 6 | open_water | 0.798 | 0.062 | 0.675 | 0.887 | 1.002 | 73000 |
| pfit[13,2] | LPS 6 | vegetation_on_by_water | 0.172 | 0.050 | 0.090 | 0.284 | 1.001 | 50000 |
| pfit[13,3] | LPS 6 | anthropogenic_shore_area | 0.022 | 0.020 | 0.004 | 0.073 | 1.002 | 2100 |
| pfit[13,4] | LPS 6 | land | 0.007 | 0.044 | 0.000 | 0.036 | 1.001 | 130000 |
| pfit[14,1] | LPS 7 | open_water | 0.868 | 0.055 | 0.772 | 0.933 | 1.024 | 11000 |
| pfit[14,2] | LPS 7 | vegetation_on_by_water | 0.119 | 0.038 | 0.058 | 0.206 | 1.001 | 15000 |
| pfit[14,3] | LPS 7 | anthropogenic_shore_area | 0.006 | 0.007 | 0.001 | 0.022 | 1.002 | 2300 |
| pfit[14,4] | LPS 7 | land | 0.007 | 0.046 | 0.000 | 0.034 | 1.001 | 50000 |

**Table S5. Full GAMM Results for activity**

**Model summary**

- Error distribution: Gaussian
- Link function: Identity
- Adjusted $R^2 = 0.147$
- Scale estimate = 2.7766
- *n* = 1,785

*A. Parametric fixed effects (log-activity scale) - activity*

| | Estimate | Std. Error | Confidence Interval (95%) | |
|---|---|---|---|---|
| | | | 2.5% | 97.5% |
| (Intercept) | 7.737 | 0.274 | 7.199 | 8.274 |
| treatmentLPS | -0.399 | 0.210 | -0.811 | 0.012 |
| tz2adm | 1.544 | 0.336 | 0.886 | 2.202 |
| tz2cda | 2.136 | 0.298 | 1.552 | 2.719 |
| tz2cdm | 1.016 | 0.426 | 0.182 | 1.851 |
| tz2d1 | 1.165 | 0.310 | 0.557 | 1.773 |
| tz2d10 | 0.256 | 0.299 | -0.331 | 0.843 |
| tz2d2 | 0.997 | 0.312 | 0.385 | 1.610 |
| tz2d3 | 0.645 | 0.316 | 0.026 | 1.264 |
| tz2d4 | 0.183 | 0.311 | -0.425 | 0.792 |
| tz2d5 | 0.357 | 0.306 | -0.243 | 0.956 |
| tz2d6 | 0.471 | 0.301 | -0.120 | 1.062 |
| tz2d7 | 0.124 | 0.304 | -0.471 | 0.719 |
| tz2d8 | 0.517 | 0.309 | -0.089 | 1.123 |
| tz2d9 | -0.158 | 0.303 | -0.751 | 0.435 |
| tz2n1 | -0.253 | 0.272 | -0.785 | 0.279 |
| tz2n10 | 0.311 | 0.306 | -0.289 | 0.910 |
| tz2n2 | -0.047 | 0.299 | -0.633 | 0.539 |
| tz2n3 | -0.035 | 0.303 | -0.628 | 0.558 |
| tz2n4 | -0.022 | 0.303 | -0.617 | 0.572 |
| tz2n5 | -0.255 | 0.307 | -0.857 | 0.347 |
| tz2n6 | -0.266 | 0.304 | -0.863 | 0.330 |
| tz2n7 | -0.898 | 0.306 | -1.497 | -0.298 |
| tz2n8 | -0.912 | 0.309 | -1.518 | -0.306 |
| tz2n9 | -0.421 | 0.304 | -1.016 | 0.174 |
| tz2nda | 0.804 | 0.338 | 0.141 | 1.466 |
| tz2ndm | 1.067 | 0.328 | 0.425 | 1.710 |

*adm - Astronomical twilight morning, cda - Civil twilight evening, cdm - Civil twilight morning, nda - Nautical twilight evening, ndm - Nautical twilight morning, n - night, d - day*
*Here, the 95% confidence intervals were calculated using confint().*

*B. Smooth terms - activity*

| | edf | Ref.df |
|---|---|---|
| s(hrs_ps):treatmentcontrol | 1.00 | 1.000 |
| s(hrs_ps):treatmentLPS | 3.70 | 3.698 |

*C. Random effects (variance components) - activity*

| Random_effect | SD | Variance | ICC |
|---|---|---|---|

| | | | |
|---|---|---|---|
| (Intercept) | 0.352 | 0.124 | 0.043 |
| Residual | 1.666 | 2.777 | NA |

*D. Correlation structure - activity*

| *Structure* | Phi |
|---|---|
| AR(1) | 0.252 |

AR(1) structure applied within individuals to account for temporal autocorrelation.

*E. Model fit statistics - activity*

| *n_observations* | n_individuals | Adjusted_R2 | logLik |
|---|---|---|---|
| 1785 | 18 | 0.147 | -3401.790 |

**Table S6. Full GAMM Results for speed**

**Model summary**

- Error distribution: Gaussian
- Link function: Identity
- Adjusted $R^2 = 0.116$
- Scale estimate = 2.1084
- *n = 4,378*

*A. Parametric fixed effects (log-speed scale) - speed*

| | Estimate | Std. Error | Confidence (95%) 2.5% | Interval 97.5% |
|---|---|---|---|---|
| (Intercept) | -3.728 | 0.143 | -4.009 | -3.447 |
| treatmentLPS | -0.222 | 0.096 | -0.410 | -0.034 |
| tz2d2 | -0.025 | 0.166 | -0.350 | 0.300 |
| tz2d3 | -0.476 | 0.188 | -0.843 | -0.108 |
| tz2d4 | -0.449 | 0.186 | -0.814 | -0.084 |
| tz2d5 | -0.427 | 0.182 | -0.784 | -0.071 |
| tz2d6 | -0.377 | 0.182 | -0.733 | -0.021 |
| tz2d7 | -0.200 | 0.180 | -0.554 | 0.153 |
| tz2d8 | -0.069 | 0.180 | -0.423 | 0.284 |
| tz2d9 | -0.233 | 0.179 | -0.585 | 0.118 |
| tz2d10 | 0.075 | 0.180 | -0.279 | 0.428 |
| tz2cda | 1.168 | 0.200 | 0.776 | 1.560 |
| tz2nda | 0.151 | 0.195 | -0.232 | 0.533 |
| tz2ada | -0.719 | 0.195 | -1.101 | -0.336 |
| tz2n1 | -0.476 | 0.179 | -0.828 | -0.125 |
| tz2n2 | -0.632 | 0.177 | -0.979 | -0.285 |
| tz2n3 | -0.450 | 0.177 | -0.796 | -0.103 |
| tz2n4 | -0.403 | 0.179 | -0.754 | -0.053 |
| tz2n5 | -0.788 | 0.178 | -1.137 | -0.439 |
| tz2n6 | -0.694 | 0.181 | -1.049 | -0.340 |
| tz2n7 | -1.042 | 0.177 | -1.388 | -0.696 |
| tz2n8 | -1.047 | 0.178 | -1.396 | -0.698 |
| tz2n9 | -0.837 | 0.178 | -1.186 | -0.487 |
| tz2n10 | -0.218 | 0.177 | -0.564 | 0.129 |
| tz2adm | 0.908 | 0.199 | 0.518 | 1.297 |
| tz2ndm | 0.784 | 0.195 | 0.401 | 1.167 |
| tz2cdm | 0.200 | 0.177 | -0.147 | 0.548 |

*adm - Astronomical twilight morning, cda - Civil twilight evening, cdm - Civil twilight morning, nda - Nautical twilight evening, ndm - Nautical twilight morning, n - night, d - day*
*Here, the 95% confidence intervals were calculated using confint().*

*B. Smooth terms - speed*

| | edf | Ref.df |
|---|---|---|
| s(hrs_ps):treatmentcontrol | 1.00 | 1 |
| s(hrs_ps):treatmentLPS | 1.00 | 1 |

*C. Random effects (variance components) - speed*

| Random_effect | SD | Variance | ICC |
|---|---|---|---|
| (Intercept) | 0.128 | 0.017 | 0.008 |
| Residual | 1.452 | 2.108 | NA |

*D. Correlation structure - speed*

| Structure | Phi |
|---|---|
| AR(1) | 0.34 |

AR(1) structure applied within individuals to account for temporal autocorrelation.

*E. Model fit statistics - speed*

| n_observations | n_individuals | Adjusted_R2 | logLik |
|---|---|---|---|
| 4378 | 18 | 0.116294 | -7582.177 |

**Table S7. Full GAMM Results for step length (distance travelled)**

**Model summary**
• Error distribution: Gaussian
• Link function: Identity
• Adjusted $R^2 = 0.108$
• Scale estimate = 2.022
• n = 3,914

*A. Parametric fixed effects (log-distance scale) - distance*

| | | | Confidence (95%) | Interval |
|---|---|---|---|---|
| | Estimate | Std. Error | 2.5% | 97.5% |
| (Intercept) | 3.377 | 0.148 | 3.087 | 3.667 |
| treatmentLPS | -0.249 | 0.095 | -0.434 | -0.064 |
| tz2d2 | -0.033 | 0.172 | -0.370 | 0.303 |
| tz2d3 | -0.492 | 0.197 | -0.878 | -0.106 |
| tz2d4 | -0.448 | 0.194 | -0.829 | -0.066 |
| tz2d5 | -0.432 | 0.190 | -0.805 | -0.060 |
| tz2d6 | -0.395 | 0.189 | -0.767 | -0.024 |
| tz2d7 | -0.231 | 0.188 | -0.599 | 0.137 |
| tz2d8 | -0.138 | 0.187 | -0.506 | 0.229 |
| tz2d9 | -0.207 | 0.187 | -0.574 | 0.159 |
| tz2d10 | 0.002 | 0.188 | -0.367 | 0.371 |
| tz2cda | 0.951 | 0.210 | 0.539 | 1.362 |
| tz2nda | 0.492 | 0.205 | 0.091 | 0.894 |
| tz2ada | -0.546 | 0.204 | -0.946 | -0.146 |
| tz2n1 | -0.397 | 0.188 | -0.766 | -0.027 |
| tz2n2 | -0.555 | 0.186 | -0.919 | -0.192 |
| tz2n3 | -0.441 | 0.186 | -0.805 | -0.077 |
| tz2n4 | -0.314 | 0.186 | -0.680 | 0.051 |
| tz2n5 | -0.711 | 0.186 | -1.076 | -0.347 |
| tz2n6 | -0.584 | 0.189 | -0.955 | -0.212 |
| tz2n7 | -0.952 | 0.185 | -1.314 | -0.589 |
| tz2n8 | -0.895 | 0.187 | -1.262 | -0.527 |
| tz2n9 | -0.802 | 0.186 | -1.167 | -0.437 |
| tz2n10 | -0.254 | 0.185 | -0.618 | 0.109 |
| tz2adm | 0.762 | 0.211 | 0.348 | 1.175 |
| tz2ndm | 0.964 | 0.205 | 0.561 | 1.366 |
| tz2cdm | 0.269 | 0.184 | -0.092 | 0.629 |

*adm - Astronomical twilight morning, cda - Civil twilight evening, cdm - Civil twilight morning, nda - Nautical twilight evening, ndm - Nautical twilight morning, n - night, d - day*
*Here, the 95% confidence intervals were calculated using confint().*

*B. Smooth terms - distance*

| | edf | Ref.df |
|---|---|---|
| s(hrs_ps):treatmentcontrol | 1.00 | 1.000 |
| s(hrs_ps):treatmentLPS | 1.00 | 1.000 |

*C. Random effects (variance components) - distance*

| Random_effect | SD | Variance | ICC |
|---|---|---|---|
| (Intercept) | 0.115 | 0.013 | 0.007 |

| | | | |
|---|---|---|---|
| Residual | 1.422 | 2.022 | NA |

*D. Correlation structure - distance*

| Structure | Phi |
|---|---|
| AR(1) | 0.362 |

AR(1) structure applied within individuals to account for temporal autocorrelation.

*E. Model fit statistics - distance*

| n_observations | n_individuals | Adjusted_R2 | logLik |
|---|---|---|---|
| 3914 | 17 | 0.108 | -6662.330 |

**Table S8. Full GAMM results for activity including sex as a fixed effect**

**Model summary**

- Error distribution: Gaussian
- Link function: Identity
- Adjusted $R^2 = 0.148$
- Scale estimate = 2.7766
- $n$ = 1,785

*A. Parametric fixed effects (log-activity scale) - activity*

| | Estimate | Std. Error | Confidence Interval (95%) | |
|---|---|---|---|---|
| | | | 2.5% | 97.5% |
| (Intercept) | 7.925 | 0.379 | 7.182 | 8.667 |
| treatmentLPS | -0.404 | 0.207 | -0.810 | 0.001 |
| sexM | -0.215 | 0.301 | -0.806 | 0.375 |
| tz2adm | 1.546 | 0.336 | 0.888 | 2.204 |
| tz2cda | 2.134 | 0.298 | 1.551 | 2.718 |
| tz2cdm | 1.020 | 0.426 | 0.186 | 1.855 |
| tz2d1 | 1.166 | 0.310 | 0.558 | 1.774 |
| tz2d10 | 0.257 | 0.299 | -0.330 | 0.844 |
| tz2d2 | 0.998 | 0.313 | 0.386 | 1.611 |
| tz2d3 | 0.645 | 0.316 | 0.026 | 1.264 |
| tz2d4 | 0.184 | 0.311 | -0.425 | 0.793 |
| tz2d5 | 0.357 | 0.306 | -0.242 | 0.957 |
| tz2d6 | 0.472 | 0.301 | -0.119 | 1.063 |
| tz2d7 | 0.125 | 0.304 | -0.470 | 0.721 |
| tz2d8 | 0.518 | 0.309 | -0.087 | 1.124 |
| tz2d9 | -0.156 | 0.303 | -0.750 | 0.437 |
| tz2n1 | -0.252 | 0.272 | -0.785 | 0.280 |
| tz2n10 | 0.312 | 0.306 | -0.288 | 0.911 |
| tz2n2 | -0.046 | 0.299 | -0.632 | 0.540 |
| tz2n3 | -0.035 | 0.303 | -0.628 | 0.558 |
| tz2n4 | -0.023 | 0.304 | -0.618 | 0.571 |
| tz2n5 | -0.255 | 0.307 | -0.857 | 0.348 |
| tz2n6 | -0.265 | 0.304 | -0.861 | 0.332 |
| tz2n7 | -0.896 | 0.306 | -1.496 | -0.297 |
| tz2n8 | -0.911 | 0.309 | -1.517 | -0.305 |
| tz2n9 | -0.421 | 0.304 | -1.016 | 0.174 |
| tz2nda | 0.805 | 0.338 | 0.142 | 1.467 |
| tz2ndm | 1.068 | 0.328 | 0.426 | 1.710 |

*adm - Astronomical twilight morning, cda - Civil twilight evening, cdm - Civil twilight morning, nda - Nautical twilight evening, ndm - Nautical twilight morning, n - night, d - day*
*Here, the 95% confidence intervals were calculated using confint().*
*The sex coefficient represents the difference between males and females, with females as the reference category. Because only two females were included in the analysis, this estimate should be interpreted cautiously.*

*B. Smooth terms - activity*

| | edf | Ref.df |
|---|---|---|
| s(hrs_ps):treatmentcontrol | 1.00 | 1.000 |
| s(hrs_ps):treatmentLPS | 3.70 | 3.703 |

*C. Random effects (variance components) - activity*

| Random_effect | SD | Variance | ICC |
|---|---|---|---|
| (Intercept) | 0.344 | 0.118 | 0.041 |
| Residual | 1.666 | 2.777 | NA |

*D. Correlation structure - activity*

| Structure | Phi |
|---|---|
| AR(1) | 0.252 |

*AR(1) structure applied within individuals to account for temporal autocorrelation.*

*E. Model fit statistics - activity*

| n_observations | n_individuals | Adjusted_R2 | logLik |
|---|---|---|---|
| 1785 | 0 | 0.148 | -3401.534 |

**Table S9. Full GAMM results for speed including sex as a fixed effect**

**Model summary**

- Error distribution: Gaussian
- Link function: Identity
- Adjusted $R^2 = 0.122$
- Scale estimate = 2.1098
- n = 4,378

*A. Parametric fixed effects (log-speed scale) - speed*

| | | | Confidence Interval (95%) | |
|---|---|---|---|---|
| | Estimate | Std. Error | 2.5% | 97.5% |
| (Intercept) | -3.439 | 0.153 | -3.738 | -3.139 |
| treatmentLPS | -0.236 | 0.063 | -0.359 | -0.112 |
| sexM | -0.318 | 0.086 | -0.486 | -0.150 |
| tz2d2 | -0.026 | 0.166 | -0.351 | 0.299 |
| tz2d3 | -0.476 | 0.188 | -0.843 | -0.108 |
| tz2d4 | -0.448 | 0.186 | -0.813 | -0.082 |
| tz2d5 | -0.426 | 0.182 | -0.782 | -0.069 |
| tz2d6 | -0.375 | 0.182 | -0.731 | -0.020 |
| tz2d7 | -0.197 | 0.180 | -0.550 | 0.157 |
| tz2d8 | -0.067 | 0.180 | -0.421 | 0.286 |
| tz2d9 | -0.233 | 0.179 | -0.585 | 0.118 |
| tz2d10 | 0.076 | 0.181 | -0.277 | 0.430 |
| tz2cda | 1.170 | 0.200 | 0.778 | 1.562 |
| tz2nda | 0.148 | 0.195 | -0.235 | 0.531 |
| tz2ada | -0.721 | 0.195 | -1.104 | -0.338 |
| tz2n1 | -0.476 | 0.180 | -0.828 | -0.124 |
| tz2n2 | -0.634 | 0.177 | -0.981 | -0.286 |
| tz2n3 | -0.453 | 0.177 | -0.800 | -0.106 |
| tz2n4 | -0.406 | 0.179 | -0.756 | -0.055 |
| tz2n5 | -0.790 | 0.178 | -1.139 | -0.440 |
| tz2n6 | -0.697 | 0.181 | -1.051 | -0.342 |
| tz2n7 | -1.048 | 0.177 | -1.394 | -0.701 |
| tz2n8 | -1.050 | 0.178 | -1.399 | -0.701 |
| tz2n9 | -0.838 | 0.178 | -1.188 | -0.488 |
| tz2n10 | -0.221 | 0.177 | -0.567 | 0.126 |
| tz2adm | 0.906 | 0.199 | 0.517 | 1.296 |
| tz2ndm | 0.782 | 0.195 | 0.399 | 1.165 |
| tz2cdm | 0.199 | 0.177 | -0.149 | 0.547 |

*adm - Astronomical twilight morning, cda - Civil twilight evening, cdm - Civil twilight morning, nda - Nautical twilight evening, ndm - Nautical twilight morning, n - night, d - day*

*Here, the 95% confidence intervals were calculated using confint().*

*The sex coefficient represents the difference between males and females, with females as the reference category. Because only two females were included in the analysis, this estimate should be interpreted cautiously.*

*B. Smooth terms - speed*

| | edf | Ref.df |
|---|---|---|
| s(hrs_ps):treatmentcontrol | 1.00 | 1 |
| s(hrs_ps):treatmentLPS | 1.00 | 1 |

*C. Random effects (variance components) - speed*

| Random_effect | SD | Variance | ICC |
|---|---|---|---|
| (Intercept) | 0.005 | ~0.000 | <0.001 |
| Residual | 1.453 | 2.110 | NA |

*D. Correlation structure - speed*

| Structure | Phi |
|---|---|
| AR(1) | 0.34 |

*AR(1) structure applied within individuals to account for temporal autocorrelation.*

*E. Model fit statistics - speed*

| n_observations | n_individuals | Adjusted_R2 | logLik |
|---|---|---|---|
| 4378 | 18 | 0.1217964 | -7577.614 |

**Table S10. Full GAMM Results for step length (distance travelled) including sex as a fixed effect**

**Model summary**

- Error distribution: Gaussian
- Link function: Identity
- Adjusted $R^2 = 0.113$
- Scale estimate = 2.022
- n = 3,914

*A. Parametric fixed effects (log-distance scale) - distance*

| | | | Confidence Interval (95%) | |
|---|---|---|---|---|
| | Estimate | Std. Error | 2.5% | 97.5% |
| (Intercept) | 3.646 | 0.160 | 3.332 | 3.960 |
| treatmentLPS | -0.257 | 0.067 | -0.388 | -0.126 |
| sexM | -0.310 | 0.092 | -0.491 | -0.130 |
| tz2d2 | -0.032 | 0.172 | -0.369 | 0.304 |
| tz2d3 | -0.488 | 0.197 | -0.874 | -0.102 |
| tz2d4 | -0.441 | 0.194 | -0.822 | -0.060 |
| tz2d5 | -0.424 | 0.190 | -0.796 | -0.051 |
| tz2d6 | -0.388 | 0.189 | -0.759 | -0.016 |
| tz2d7 | -0.223 | 0.188 | -0.591 | 0.145 |
| tz2d8 | -0.132 | 0.187 | -0.499 | 0.235 |
| tz2d9 | -0.202 | 0.187 | -0.569 | 0.164 |
| tz2d10 | 0.008 | 0.188 | -0.361 | 0.376 |
| tz2cda | 0.958 | 0.210 | 0.547 | 1.370 |
| tz2nda | 0.496 | 0.205 | 0.094 | 0.898 |
| tz2ada | -0.540 | 0.204 | -0.940 | -0.140 |
| tz2n1 | -0.390 | 0.188 | -0.760 | -0.021 |
| tz2n2 | -0.553 | 0.186 | -0.917 | -0.190 |
| tz2n3 | -0.439 | 0.186 | -0.803 | -0.075 |
| tz2n4 | -0.313 | 0.186 | -0.678 | 0.052 |
| tz2n5 | -0.710 | 0.186 | -1.075 | -0.346 |
| tz2n6 | -0.583 | 0.189 | -0.954 | -0.212 |
| tz2n7 | -0.955 | 0.185 | -1.317 | -0.592 |
| tz2n8 | -0.895 | 0.187 | -1.262 | -0.527 |
| tz2n9 | -0.801 | 0.186 | -1.166 | -0.437 |
| tz2n10 | -0.256 | 0.185 | -0.620 | 0.107 |
| tz2adm | 0.760 | 0.211 | 0.346 | 1.174 |
| tz2ndm | 0.966 | 0.205 | 0.563 | 1.368 |
| tz2cdm | 0.270 | 0.184 | -0.090 | 0.631 |

*adm - Astronomical twilight morning, cda - Civil twilight evening, cdm - Civil twilight morning, nda - Nautical twilight evening, ndm - Nautical twilight morning, n - night, d - day*

*Here, the 95% confidence intervals were calculated using confint().*

*The sex coefficient represents the difference between males and females, with females as the reference category. Because only two females were included in the analysis, this estimate should be interpreted cautiously.*

*B. Smooth terms - distance*

| | edf | Ref.df |
|---|---|---|
| s(hrs_ps):treatmentcontrol | 1.00 | 1.000 |
| s(hrs_ps):treatmentLPS | 1.00 | 1.000 |

*C. Random effects (variance components) - distance*

| Random_effect | SD | Variance | ICC |
|---|---|---|---|
| (Intercept) | 0.001 | <0.001 | <0.001 |
| Residual | 1.422 | 2.022 | NA |

*D. Correlation structure - distance*

| Structure | Phi |
|---|---|
| AR(1) | 0.362 |

*AR(1) structure applied within individuals to account for temporal autocorrelation.*

*E. Model fit statistics - distance*

| n_observations | n_individuals | Adjusted_R2 | logLik |
|---|---|---|---|
| 3914 | 17 | 0.113 | -6658.178 |

**Table S11**. The posterior probability (certainty) of the hypothesis that the mean proportional use of a habitat type is smaller in the control group than in the treatment group from the first until the seventh days post stimulation (dps). Results from the multinomial model when using the land cover map from R.T.K. (results shown in manuscript).

| habitat class / days post stimulation (dps) | 1 dps | 2 dps | 3 dps | 4 dps | 5 dps | 6 dps | 7 dps |
|---|---|---|---|---|---|---|---|
| open water | 0.024 | 0.010 | 0.152 | 0.850 | 0.989 | 0.991 | 0.998 |
| (semi)natural vegetation close to or on water | 0.976 | 0.959 | 0.771 | 0.144 | 0.017 | 0.013 | 0.134 |
| anthropogenic shore area | 0.675 | 0.843 | 0.787 | 0.663 | 0.863 | 0.700 | 0.003 |
| land/farmland | 0.222 | 0.863 | 0.575 | 0.246 | 0.035 | 0.066 | 0.020 |

**Table S12**. The posterior probability (certainty) of the hypothesis that the mean proportional use of a habitat type is smaller in the control group than in the treatment group from the first until the seventh days post stimulation (dps). Results from the multinomial model when using the land cover map from E.J. (results shown for comparison to those in main manuscript).

| habitat class / days post stimulation (dps) | 1 dps | 2 dps | 3 dps | 4 dps | 5 dps | 6 dps | 7 dps |
|---|---|---|---|---|---|---|---|
| open water | 0.031 | 0.011 | 0.188 | 0.850 | 0.993 | 0.995 | 0.998 |
| (semi)natural vegetation close to or on water | 0.969 | 0.951 | 0.728 | 0.131 | 0.011 | 0.009 | 0.097 |
| anthropogenic shore area | 0.457 | 0.851 | 0.784 | 0.672 | 0.869 | 0.792 | 0.003 |
| land/farmland | 0.222 | 0.866 | 0.583 | 0.297 | 0.052 | 0.056 | 0.016 |

# Supplementary text

**Supplementary Text S1. Avian influenza screening**

To evaluate if any of the mallards included in our experiment were already fighting an infection, we screened all individuals for avian influenza virus. For this purpose, we collected oral and cloacal swabs at the time of capture. We placed the swabs in Sigma-Virocult® virus transport medium and stored them at -80°C within 1 to 2 h after collection. We extracted RNA from the swabs as described previously[1]. Briefly, RNA was isolated using Tri Reagent BD (Sigma) supplemented with acetic acid. We then added the aqueous phase from the phase separation step in the Tri Reagent manual to a new tube, and processed the samples as described in step 4 (Load and Bind) and onwards in the peqGOLD Blood RNA Kit manual. We used the DNase I Digest Kit from peqGOLD to remove contaminating DNA.

The samples were then screened for avian influenza virus by the National Reference Laboratory for Avian Influenza (AI) in Germany, at the Institute of Diagnostic Virology (IVD) at the Friedrich-Loeffler-Institut (https://www.fli.de/en/institutes/institute-of-diagnostic-virology-ivd/reference-laboratories/woah-fao-and-nrl-for-ai/). All individuals tested negative for AI infection.

1 Jax E et al. 2021 Health monitoring in birds using bio-loggers and whole blood transcriptomics. Sci. Rep. 11, 10815. (doi:10.1038/s41598-021-90212-8)
